# Molecular and Functional Characterization of the Somatic PIWIL1/piRNA Pathway in Colorectal Cancer Cells

**DOI:** 10.3390/cells8111390

**Published:** 2019-11-05

**Authors:** Assunta Sellitto, Konstantinos Geles, Ylenia D’Agostino, Marisa Conte, Elena Alexandrova, Domenico Rocco, Giovanni Nassa, Giorgio Giurato, Roberta Tarallo, Alessandro Weisz, Francesca Rizzo

**Affiliations:** 1 Laboratory of Molecular Medicine and Genomics, Department of Medicine, Surgery and Dentistry “Scuola Medica Salernitana”, University of Salerno, 84081 Baronissi (SA), Italy; assellitto@unisa.it (A.S.); yeles.konstantinos@gmail.com (K.G.); ydagostino@unisa.it (Y.D.); marisaconte30@gmail.com (M.C.); ealexandrova@unisa.it (E.A.); drocco@unisa.it (D.R.); gnassa@unisa.it (G.N.); ggiurato@unisa.it (G.G.); rtarallo@unisa.it (R.T.); 2 Genomix4Life srl, Department of Medicine, Surgery and Dentistry “Scuola Medica Salernitana”, University of Salerno, 84081 Baronissi (SA), Italy; 3 Medical Genomics Program, Department of Onco-Haematology SS. Giovanni di Dio e Ruggi d’Aragona’ University Hospital, University of Salerno, 84131 Salerno, Italy

**Keywords:** colorectal cancer, small non-coding RNAs, RNA-binding proteins, PIWIL1, piRNAs, nuage compartment, RIP-Seq

## Abstract

**PIWI-like** (PIWIL) proteins and small non-coding piRNAs, involved in genome regulation in germline cells, are found aberrantly expressed in human tumors. Gene expression data from The Cancer Genome Atlas (TCGA), the Genotype-Tissue Expression (GTEx) project, and the European Genome-Phenome Archive (EGA) indicate that the *PIWIL1* gene is ectopically activated in a significant fraction of colorectal cancers (CRCs), where this is accompanied by promoter demethylation, together with germline factors required for piRNA production. Starting from this observation, the PIWIL/piRNA pathway was studied in detail in COLO 205 CRC cells, which express significant levels of this protein, to investigate role and significance of ectopic *PIWIL1* expression in human tumors. RNA sequencing and cell and computational biology led to the demonstration that PIWIL1 localizes in a nuage-like structure located in the perinuclear region of the cell and that a significant fraction of the piRNAs expressed in these cells are methylated, and, therefore, present in an active form. This was further supported by RNA immunoprecipitation, which revealed how several piRNAs can be found loaded into PIWIL1 to form complexes also comprising their target mRNAs. The mature transcripts associated with the PIWIL–piRNA complex encode key regulatory proteins involved in the molecular mechanisms sustaining colorectal carcinogenesis, suggesting that the PIWI/piRNA pathway may actively contribute to the establishment and/or maintenance of clinico-pathological features of CRCs.

## 1. Introduction

Cancer and germ cells share essential similarities, such a high proliferation rate and self-renewal ability, and cancer cells sometimes re-activate cancer testis antigen (CTA) genes, whose expression is usually restricted to the germline and silenced in adult somatic tissues. The expression of germline genes in cancer may reflect the aberrant activation of a silenced developmental program that leads to the escape from cell death, immune evasion, and invasiveness, thus contributing to the molecular mechanisms of carcinogenesis [1]. Among CTAs, *PIWI-like* (*PIWIL*) genes, belonging to the Argonaute family, have recently attracted attention as they are frequently deregulated in cancer and have been associated with advanced tumor stage and poor prognosis, making them useful biomarkers for cancer diagnosis and possible druggable targets [2,3].

The PIWI-like family includes evolutionarily conserved proteins (PIWIL1-4) with key functions in the regulation of gene expression [4], whose activity is mediated by the association with a specific class of small non-coding RNAs (sncRNAs) of 20–35 nt in length, defined as PIWI-interacting RNAs (piRNAs). In non-pathological conditions, both PIWILs and piRNAs are abundantly expressed in the germline, where they are involved in transposable element silencing [5]. However, several studies revealed that not only transposon transcripts but also mRNAs are targeted by piRNAs, by binding to their 3’UTR and the induction of mRNA degradation [6], mainly during embryogenesis and germ-cell development [7]. Recently, a crucial role for the PIWIL–piRNA complex has also emerged in somatic cells, where growing evidence indicates that both PIWIL and piRNA expression can be reactivated under pathological stimuli [8,9,10,11]. In this respect, in vitro/in vivo studies demonstrated that PIWIL proteins are not only a marker of malignancy but are also involved in cell cycle regulation, tumorigenesis, drug resistance, and acquisition of self-renewal abilities [12,13].

Colorectal (CRC) is one of the most common human cancers, and still the third leading cause of cancer-related mortality. Identifying new diagnostic and therapeutic targets for CRC therefore remains a priority in cancer research. Interestingly, in the last few years, piRNAs emerged as promising diagnostic and prognostic biomarkers for CRC [14,15,16], where ectopic presence of PIWIL1 has recently been correlated with tumor differentiation status, infiltration, lymph node invasion and metastasis [17,18,19]; patients with PIWIL1 overexpression also showed lower overall survival and disease-free survival [20]. Moreover, the expression of PIWIL1 in CRC has been positively correlated with the mRNA level of OCT4, a cancer stem cell marker, indicating that this gene may contribute to the tumor stemless, which in turn is strongly related to its metastatic potential [21]. However, the molecular mechanisms and biological roles of PIWILs and piRNAs have not been explored in detail, so that the cellular pathways and molecular mechanisms in which somatic piRNAs are involved are still poorly known.

We thus investigated here the co-expression and possible functional relationship between PIWILs and piRNAs in CRC. To this end, the PIWIL1/piRNA pathway was characterized in CRC tumor tissues and cell lines, combining RNA sequencing, methylation data, and protein biochemistry. Interestingly, we observed in human CRC cells that PIWIL1 localizes in nuage-like perinuclear granules in the cytoplasmic perinuclear region of the cell, a result that, combined with the identification of mature piRNAs carrying a methyl group at their 3′-end, indicates the presence of an active piRNA pathway in CRC cells. Finally, we demonstrated, in COLO 205 cells, the physical association between PIWIL1 and several piRNAs and their predicted mRNA targets, a result experimentally validated by RNA immunoprecipitation (RIP)-Seq. The mature transcripts associated with the PIWIL–piRNA complex are involved in the control of cell proliferation, differentiation, and survival in cancer cells. These data demonstrate that re-activation of this germline pathway in CRC cells can contribute to the regulation of cellular pathways controlling key functions of the cancerous cell.

## 2. Materials and Methods

### 2.1. Gene Expression Databases and DNA Methylation Data Analysis

The expression level of 4 *PIWIL* genes was evaluated across 31 tumor types listed in the PanCancer Atlas [22] by mining RNA-Seq data from two databases, The Cancer Genome Atlas (TCGA) and the Genotype-Tissue Expression (GTEx). Expression bar plots for *PIWIL* genes were obtained using the Gepia2 web server [23]. Transcriptomic data for 53 CRC cell lines were downloaded from the European Genome-Phenome Archive (EGA, https://ega-archive.org/) dataset (E-MTAB-2706). To evaluate the relationship between *PIWIL1* expression and DNA methylation of tumor tissues, Infinium HumanMethylation450 data and RNA-Seq data for 275 colon adenocarcinomas and 19 normal tissues (TCGA data) were downloaded from the TCGA database. For the methylation analysis of 8 CRC cell lines, Infinium HumanMethylation450 collected by Barault el al. (GEO accession GSE86078) were used [24].

### 2.2. Cell Culture

Human colon cancer cell lines Caco-2, SW403, SW1417, COLO 205, HT-29, HCT 116, and RKO were supplied by the American Type Culture Collection (ATCC, Rockville, MD, USA); the HT115 cell line was obtained from Sigma-Aldrich, Milan, Italy. All cell lines were cultured following the manufacturer’s instructions; culture media were supplemented with fetal bovine serum (HyClone, Cramlington, UK), 100 U/mL penicillin, 100 mg/mL streptomycin, and 250 ng/mL Amphotericin-B. The identity of all cell lines was confirmed by short tandem repeat (STR) profiling; cells were routinely screened for mycoplasma contamination with MycoAlert mycoplasma detection kit (Lonza, Milan, Italy).

### 2.3. Transient Transfections

For the transient overexpression of PIWIL1, HCT 116 cells were transfected with the human full-length cDNA clone pCMV6-PIWIL1 (RC205269) or with control pCMV6-Entry mammalian vector (PS100001) (Origene, Herford, Germany). At 24 h prior to transfection, HCT 116 cells at the exponential growth phase were seeded in 100 mm culture dishes; the next day, plates at 60% confluency were washed and re-fed with culture medium shortly before transfection. A total of 15 µg of DNA was mixed with linear 25 kDa polyethyleneimine (PEI) (Polysciences, Eppenheim, Germany) and incubated for 20 min at room temperature, then the DNA/PEI mixture was added to plates. After 24 h from transfection, HCT 116-pCMV6-PIWIL1 cells were analyzed by immunofluorescence using rabbit anti-PIWIL1 (ab12337, Abcam, Cambridge, UK); fluorescence images were collected with a LEICA DM6000 B Confocal Microscope and used to evaluate and quantify transfection efficiency, which was found to be ~20%.

### 2.4. Real-Time qRT-PCR

To generate cDNA with the AffinityScript cDNA Synthesis Kit (Agilent Technologies, Rome, Italy), 1 μg of total RNA was used. cDNAs were diluted to a final concentration of 20 ng per reaction. Real-time qRT-PCR was performed in triplicate using Brilliant II SYBR Master Mixes (Agilent Technologies) on an Mx3005P Instrument (Agilent Technologies); the expression level of *PIWIL* genes was normalized against β-actin mRNA. Specific primer sets are reported in Table 1.

### 2.5. Total Protein Extraction

Cells at exponential growth phase were harvested by scraping in cold phosphate buffered saline (PBS) and collected by centrifugation at 1000× *g*. Then, for total protein extraction, pelleted cells were resuspended in 3 volumes with respect to the cell pellet of hypotonic buffer (50 mM Tris-HCl, 0.15% NP40, 10% Glycerol, 180 mM NaCl, 1.5 mM MgCl2, 1 mM NaMoO4, 0.5 mM NaF) supplemented with 1× Protease Inhibitor Cocktail (Sigma-Aldrich, Milan, Italy), 1 mM DTT and 1 mM PMSF. Upon incubation on ice for 15 min, cell lysate was clarified by centrifugation at 20,000× *g* for 15 min at 4 °C, then the salt concentration was adjusted with hypotonic buffer without salts addition (2V with respect to the hypotonic buffer previously added).

### 2.6. Cytosol/Nucleus Protein Fractionation

For cytosol/nucleus protein fractionation, pelleted cells were resuspended in 3 volumes with respect to the cell pellet of hypotonic buffer (20 mM HEPES pH 7.4, 5 mM NaF, 10 μM NaMoO_4_, 0.1 mM EDTA) supplemented with 1× Protease Inhibitor Cocktail (Sigma-Aldrich, Milan, Italy), 1 mM DTT and 1 mM PMSF; upon incubation on ice for 15 min, 0.5% Triton X-100 was added, and lysate was centrifuged at 15,000× *g* for 30 s at 4 °C in order to collect the cytosolic fraction, which was further clarified by centrifugation at 15,000× *g* for 15 min. The nuclear pellets were first stratified in sucrose gradient to remove cytosolic contaminants and then resuspended in 1 volume of nuclear lysis buffer (20 mM HEPES, pH 7.4, 25% glycerol, 420 mM NaCl, 1.5 mM MgCl_2_, 0.2 mM EDTA) supplemented with 1× Protease Inhibitor Cocktail (Sigma-Aldrich, Milan, Italy), 1 mM DTT, and 1 mM PMSF; nuclei were incubated for 30 min at 4 °C with gentle shaking and then centrifuged at 15,000× *g* for 30 min at 4 °C. Salt concentration was adjusted with 1 volume of nuclear lysis buffer without salt addition, supplemented with 1× Protease Inhibitor Cocktail (Sigma-Aldrich, Milan, Italy), 1 mM DTT, and 1 mM PMSF.

### 2.7. Cytosol/Perinucleus/Nucleus Protein Fractionation

Protein fractionation was performed as previously described by Shaiken et al. [25]. Briefly, cells at exponential growth phase were harvested by scraping in cold PBS, collected by centrifugation at 1000× *g*, and resuspended in 3 volumes with respect to the cell pellet of isotonic buffer A (40 mM HEPES pH 7.4, 120 mL KCl, 2 mM EGTA, 0.4% Glycerol, 10 mM β-glycerophosphate, and 0.4% NP-40) supplemented with 1× Protease Inhibitor Cocktail (Sigma-Aldrich, Milan, Italy), 1 mM DTT, and 1 mM PMSF; cells were incubated under gentle rotation for 30 min at 4 °C, then nuclei were pelleted by centrifugation at 1000× *g* for 5 min; the supernatant containing the cytosolic fraction was clarified at 10,000× *g* for 10 min; nuclei were gently washed two times (first with 0.1% NP-40 and then with no-detergent-containing Buffer A), then centrifuged at 1000× *g* for 5 min. To isolate the perinuclear fraction, the nuclear pellet was resuspended in 1 volume of Buffer B (10 mM Tris-HCl pH 7.4, 1.5 mM KCl, 0.5% Triton X-100; 0.5% deoxycholate, 2.5 mM MgCl_2_) supplemented with fresh 0.2 M LiCl, 1× Protease Inhibitor Cocktail (Sigma-Aldrich, Milan, Italy), 1 mM DTT, and 1 mM PMSF; after 1 h of incubation at 4 °C under gentle rotation, the extract was centrifuged at 10,000× *g* for 10 min to collect the perinuclear fraction; nuclei were resuspended in 0.34 M sucrose and centrifuged at 2000× *g* for 10 min in order to remove contaminants; nuclei were finally lysed in 8 M urea and sonicated 3 times (30 sec ON, 30 sec OFF); the nuclear extract was further clarified by centrifugation at 10,000× *g* for 10 min.

### 2.8. SDS-Page and Western Blotting (WB)

Protein expression was analyzed by SDS page, in each case loading 30 µg of protein extract, and western blotting (WB) using standard protocols and the following primary antibodies: rabbit anti-PIWIL1 (ab105393, Abcam, Cambridge, UK), rabbit anti-Lamin B2 (ab151735, Abcam, Cambridge, UK), rabbit anti-DDX6 (ab40684, Abcam, Cambridge, UK), mouse anti-β-Actin (ab6276, Abcam, Cambridge, UK), mouse anti-β-Tubulin (T6074, Sigma-Aldrich, Milan, Italy). The specificity of anti-PIWIL1 antibody was tested against the transfected protein on HCT 116 cell lysate.

### 2.9. Immunofluorescence (IF) Assay and Confocal Microscopy

For IF, cells were fixed with 4% paraformaldehyde for 20 min and washed with PBS-Tween; permeabilization was performed in 0.1% Triton-X-100 in PBS for 10 min, then cells were incubated in blocking buffer (0.5% BSA in PBS-Tween) for 1 h. Slides were incubated at 4 °C overnight with rabbit anti-PIWIL1 (ab12337, Abcam, Cambridge, UK). The next day, slides were washed with 0.5% BSA-PBS-Tween and incubated for 45 min at room temperature with Alexa Fluor 488 goat anti-rabbit IgG (Thermo-Fisher, Carlsbad, CA) secondary antibody in PBS. For nuclei staining, 4′,6-diamidino-2-phenylindole (DAPI, Sigma Aldrich, St. Louis, MS, USA) was used. Images were collected with a Leica DM6000 B confocal microscope and processed with ImageJ software (https://imagej.net).

### 2.10. Sodium-Periodate (NaIO_4_) Treatment/β-Elimination

For the sodium periodate treatment, 20 µg of RNA were mixed with borate buffer pH 8.6 (29.6 mM Na_2_B_4_O_7_ 10 H_2_O; 29.6 mM H_3_BO_3_) and 25 mM NaIO_4_, then the reaction was incubated for 40 min at 4 °C in the dark; to quench the unreacted NaIO_4_, glycerol was added (10% final) and samples were incubated for 30 min at 4 °C; then, RNA was ethanol-precipitated and resuspended in RNAse-free water. For the β-elimination step, borate buffer pH 9.5 (29.6 mM Na_2_B_4_O_7_ 10 H_2_O; 29.6 mM H_3_BO_3_) was added and samples were incubated for 90 min at 45 °C; then, RNA was ethanol-precipitated and resuspended in RNAse-free water. The experiment was performed in triplicate. To monitor the efficiency of the reaction, single-stranded (SS) spike-in molecules were chemically synthesized at Exiqon, adapting the sequences described in Locati et al. [26] to our conditions. Two non-methylated (SS-22 and SS-28) and two methylated (mSS-22 and mSS-28) of 22 nt and 28 nt were used:

SS-22: 5′-UAUCAGUGCUACGUGUCUCAGU-3′

SS-28: 5′-CGUAUCGCUGCUCUGAGUCACUAUCUAC-3′

mSS-22: 5′-AUGCUGAUGAUAGACGCUACUmG-3′

mSS-28: 5′-AGAUAGUACUGAUCUGCUGCGACGAGUmG-3′

Spike-ins were pooled in equimolar concentration, then a final amount of 0.3 × 10^10^ molecules/µg of RNA was added to each sample before the treatment.

### 2.11. RNA Extraction and Sequencing with Illumina Technologies

Total RNA extraction from cell lines was performed using TRIzol reagent (Invitrogen, Carlsbad, Canada) following the manufacturer’s instructions. For small RNA sequencing, indexed libraries were prepared using 1 μg of total RNA with a TruSeq small RNA Sample Prep Kit (Illumina, San Diego, Canada). As a control for germline piRNAs, a human testis RNA pool (Biochain, Newark, Canada) was included in all experiments.

Equimolar concentration of each library was sequenced for 75 cycles on the NextSeq 500 platform (Illumina, San Diego, CA, USA). For RNA sequencing, 1 μg of total RNA was used for library preparation with a TruSeq Stranded Total RNA Sample Prep Kit (Illumina, San Diego, CA, USA) and sequenced at a concentration of 8 pM/lane (paired-end, 2 × 100 cycles) on the HiSeq 2500 platform (Illumina, San Diego, CA, USA). All sequencing experiments were performed at Genomix4life, Italy (https://www.genomix4life.com/).

### 2.12. RNA Immunoprecipitation and Sequencing (RIP-Seq)

RNA immunoprecipitation of PIWIL1-associated small RNAs was performed in biological triplicate. Briefly, COLO 205 cells were lysed in polysome lysis buffer as described by Keene et al., 2006 [27], then an aliquot of extract corresponding to 1 × 10^6^ cells was taken as input, while 8 mg of protein lysate were mixed with one of the following antibodies: rabbit anti-PIWIL1 (ab105393, Abcam, Cambridge, UK), rabbit anti-PIWIL1 (ab12337, Abcam, Cambridge, UK), rabbit IgG isotype control (02-6102, Thermo Fisher Scientific, Carlsbad, Canada), or without primary antibody addiction (No Antibody Control Beads). After overnight incubation, Dynabeads M-280 Sheep Anti-Rabbit IgG (11203-D, Thermo Fisher Scientific, Carlsbad, Canada) were added and the mixture was incubated again for 3 h at 4 °C with gentle rotation. After binding, 1/10 of each sample was taken for WB analysis, while 9/10 were used for the isolation of PIWIL1 co-precipitated RNAs with the mirVana PARIS Isolation Kit (AM1556, Ambion Inc., Austin, TX, USA). For small RNA sequencing analyses, 500 ng of RNA were used for indexed sequencing library preparation with a TruSeq small RNA Sample Prep Kit (Illumina, San Diego, CA, USA).

### 2.13. Bioinformatic Data Analysis

To identify small RNA sequences, preprocessing, alignment, and annotation to small RNAs were performed with the SPORTS1.0 pipeline [28], with the only difference that miRBase was updated to 22.1 and piRBase to the 2.0 release. In brief, SPORTS1.0 performs sequential mapping against the reference genome, miRBase, rRNA, GtRNAdb, Ensembl, Rfam, and finally, piRBase. Unannotated sequences matching to the reference genome were extracted from the final resulting file that includes all reads. The complete list of small RNA databases used, the details of small RNA and methylated piRNA identification, the strategy used to identify PIWIL1-bound piRNAs in the RIP-Seq experiment, and the functional enrichment and pathway analysis are reported in the Supplementary Methods. piRNA target prediction was performed with iSmaRT [29].

### 2.14. Availability of Supporting Data

Sequencing data were deposited in the EBI ArrayExpress database (http://www.ebi.ac.uk/arrayexpress) with the following Accession Numbers: E-MTAB-8115, E-MTAB-8116, E-MTAB-8117, and E-MTAB-8121.

## 3. Results

### 3.1. The Germline-Specific PIWIL1 Gene is Aberrantly Expressed in Colorectal Adenocarcinomas

**Figure 1:**
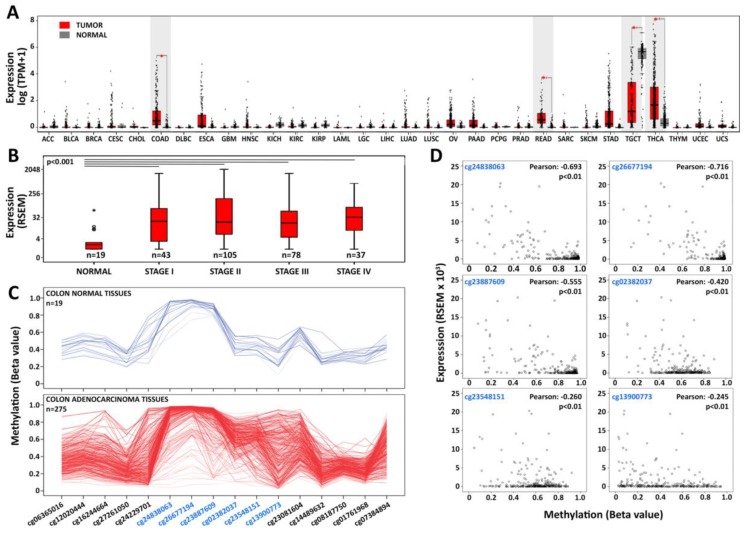
The germline-specific *PIWIL1* gene is ectopically activated in colorectal cancer. (**A**) Integrative analysis of The Cancer Genome Atlas (TCGA) and the Genotype-Tissue Expression GTEx data showed that *PIWIL1*, abundantly expressed in the germline (TCGC, normal), is aberrantly activated in cancer and significantly upregulated (highlighted in grey) in colon (COAD), rectum (READ), and thyroid carcinomas (THCA) with respect to the normal tissue (log_2_ (median (Tumor)–median (Normal)) > 0.5; *p* value < 0.01). Expression levels are displayed in log_2_-transformed (TPM (transcripts per million) + 1). ACC: adrenocortical carcinoma; BLCA: bladder urothelial carcinoma; BRCA: breast invasive carcinoma; CESC: cervical squamous cell carcinoma and endocervical adenocarcinoma; CHOL: cholangio carcinoma; COAD: colon adenocarcinoma; DLBC: lymphoid neoplasm diffuse large B-cell lymphoma; ESCA: esophageal carcinoma; GBM: glioblastoma multiforme; HNSC: head and neck squamous cell carcinoma; KICH: kidney chromophobe; KIRC: kidney renal clear cell carcinoma; KIRP: kidney renal papillary cell carcinoma; LAML: acute myeloid leukemia; LGG: brain lower grade glioma; LIHC: liver hepatocellular carcinoma; LUAD: lung adenocarcinoma; LUSC: lung squamous cell carcinoma; OV: ovarian serous cystadenocarcinoma; PAAD: pancreatic adenocarcinoma; PCPG: pheochromocytoma and paraganglioma; PRAD: prostate adenocarcinoma; READ: rectum adenocarcinoma; SARC: sarcoma; SKCM: skin cutaneous melanoma; STAD: stomach adenocarcinoma; TGCT: testicular germ cell tumor; THCA: thyroid carcinoma; THYM: thymoma; UCEC: uterine corpus endometrial carcinoma; UCS: uterine carcinosarcoma. (**B**) Boxplots showing the expression of *PIWIL1* across different AJCC (American Joint Committee on Cancer) stages of TCGA colon adenocarcinomas. (**C**) DNA methylation profile of CpGs in the promoter region of *PIWIL1* in colon normal tissues (in blue) and adenocarcinomas (in red); CpG sites highlighted in blue constitute a CpG island and are strongly methylated in colon normal tissues, while lower methylation levels are observed in several tumors. (**D**) Statistically significant negative correlation between *PIWIL1* expression and DNA methylation at CpG sites hypomethylated in tumors; beta values are plotted on the *x*-axis and RSEM (RNA-Seq by expectation-maximization) gene expression values on the *y*-axis.

To investigate the expression of *PIWIL1* in human normal and cancerous tissues, we mined RNA-Seq data in The Cancer Genome Atlas (TCGA) and/or the Genotype-Tissue Expression (GTEx) databases, including 9736 tumoral (T) and 8587 non-tumoral (N) samples organized in 31 cohorts. Results showed that in non-pathological tissues, *PIWIL1* is almost exclusively detectable in the germline (Figure 1A). In tumors, instead, this gene is significantly up-regulated (FC > 1.5; *p* value < 0.05), compared to matched normal tissues, in colon adenocarcinoma (COAD), rectum adenocarcinoma (READ), and thyroid carcinomas (THCA) when considering both dataset (Figure 1A), or in COAD and THCA in the TCGA data (Figure S1A).

On the other hand, *PIWIL2* and *PIWIL4* show low expression in many normal tissues and are upregulated in several cancer types, while *PIWIL3* is almost undetectable in all tissues considered, except germline (Figure S1). Moreover, the expression of *PIWIL1* seems to represent an early event in CRC carcinogenesis since the gene is significantly upregulated here (*p* value < 0.001), compared to normal tissues, starting from the earliest stages (I–II) of CRC progression (Figure 1B). DNA methylation, histone modifications, and chromatin remodeling are important epigenetic regulators of gene expression. Among them, however, an aberrant DNA methylation pattern is a hallmark event in colorectal carcinogenesis, characterized by either global hypomethylation or paradoxical gene-specific hypermethylation [30], which in turn drive neoplastic transformation and cancer progression via oncogene activation and tumor suppressor gene silencing. It has been shown that also *PIWIL1* expression may be linked to its epigenetic asset since, DNA hypermethylation at CpG islands located in the promoter region and first exon of the gene has been verified to occur during germ cell development and to be maintained in somatic cells, all conditions where its expression is not anymore required [31]. CpG island methylation is also the mechanism for *PIWIL1* down-regulation in testicular tumors [32]. Interestingly, we noticed a relationship between *PIWIL1* expression and DNA methylation. Analyzing DNA methylation data from 275 colon adenocarcinoma and 19 normal cases from TCGA, a group of CRC tissues (~14%) shows loss of DNA methylation in a CpG island in the promoter region of *PIWIL1* (Figure 1C, Table S1A). Specifically, we identified 6 CpG sites (cg24838063, cg26677194, cg23887609, cg02382037, cg23548151, cg13900773) in the CpG island located in the promoter and first exon of *PIWIL1*, strongly demethylated in cancers. Moreover, we observed a significant negative correlation (*p* value < 0.01 and Pearson correlation < −0.2) between *PIWIL1* promoter methylation involving all six CpGs and *PIWIL1* mRNA levels (Figure 1D). Loss of DNA methylation and re-expression is a peculiar feature of cancer testis antigens (CTAs), germline-specific genes that are normally downregulated in somatic differentiated tissues. Interestingly, *PIWIL1* has been listed among very highly expressed cancer testis genes (EECTGs), considered epigenetic drivers of cancer as when expressed, they confer a selective growth advantage to the transformed cell; they therefore may represent potential targets for new cancer therapies [33]. In line with these findings, Xie et al. [34] recently reported a similar mechanism for *PIWIL1* re-expression in lung cancer, where gene re-activation is attributed to DNA hypomethylation. Several studies explored the effects of DNA methylation inhibitors, such as 5-aza-2-deoxycytidine (5-AZA-CdR), and on gene reactivation in cancer, and two studies have investigated, in particular, their effects in several CRC cell lines [30,35]. We analyzed the results of these studies focusing on *PIWIL1* and found that in Caco2, HCT 116, HT29, and RKO cells, the promoter of this gene is not demethylated by treatment with 5-AZA-CdR, and, consequently, expression of the corresponding mRNA is not enhanced ([30,35] and data not shown). On the other hand, Yagi et al. showed a slightly positive effect on *PIWIL1* expression of a combined treatment with 5-AZA-CdR and Trichostatin A (TSA, a histone deacetylase inhibitor) in HCT 116 cells, while upregulation of the gene was observed using only 5-AZA-CdR in the SW480 cell line, which already expresses this gene at low levels ([27] and data not shown). Based on these results, it is not possible to exclude that *PIWIL1* silencing might involve a combination of DNA methylation and repressive histone modifications.

### 3.2. PIWIL and piRNA Expression in CRC Cell Lines

Interestingly, a recent genome-wide CRISPR–Cas9 loss-of-function genetic screening, prompted by the Achilles project of the Broad Institute to create a catalog of genes essential for cell survival [36], showed that *PIWIL1* knockout has an effect on survival in 55 cell lines from different tumor types, including CRC (Supplementary Table S2).

**Figure 2:**
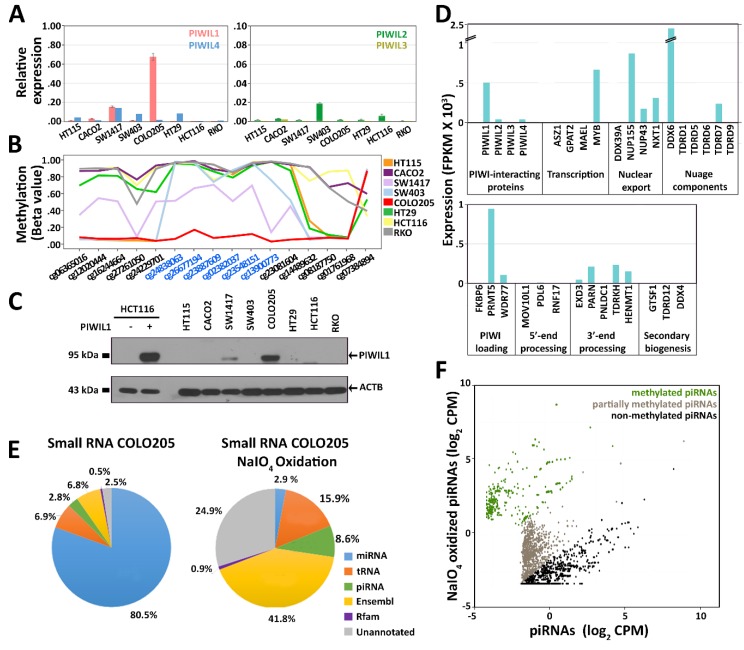
Characterization of main molecular features of the piRNA pathway in colorectal cancer (CRC) cell lines. (**A**) The expression level of *PIWIL* mRNAs in 8 CRC cell lines was determined by real-time qRT-PCR. The highest expression for *PIWIL1* was observed in COLO 205 and SW-1417. *PIWIL2* and *PIWIL4* were identified in some cell lines but at lower levels, while *PIWIL3* was undetectable in all cases. (**B**) DNA methylation profile of *PIWIL1* promoter in 8 CRC cell lines; a marked hypomethylation, correlated to *PIWIL1* expression, was observed for two cell lines (COLO 205 and SW-1417), while a strong methylation was present in the absence of *PIWIL1*. CpG sites highlighted in blue constitute a CpG island. (**C**) Western blotting analysis performed onto total protein lysate from 8 CRC cell lines confirmed the presence of abundant PIWIL1 levels in COLO 205 and medium-abundant levels in SW-1417; as a control to test the specificity of the antibody, total protein lysate from HCT 116 cells overexpressing PIWIL1 or transfected with empty vector were loaded in the same gel; β-actin was used as a loading control. (**D**) Gene expression (indicated as fragment per kilobase million, FPKM) was measured for known germline components of the piRNA pathway genes by RNA-Seq; genes were grouped according to their role in the piRNA pathway. (**E**) Pie-chart representing the relative abundance of small RNA categories identified in non-treated (left) and sodium-periodate (NaIO_4_)/β-elimination oxidized RNA (right) from COLO 205; small RNA-Seq analysis confirmed the presence of piRNAs in the COLO 205 cell line and their resistance to the sodium periodate/β-elimination treatment. (**F**) Scatterplot representing the effect of the sodium periodate (NaIO_4_)/β-elimination treatment on the piRNAs population; log_2_ CPM (count per million) values are displayed per each COLO 205 piRNA in treated (*y*-axis) and non-treated (*x*-axis) RNA; the treated (T) vs. non-treated (NT) log_2_ ratio of expression values (*p* value < 0.01) was used to identify 767 methylated piRNAs (log_2_(T/NT) > 2), 1514 partially methylated piRNAs (−2 < log_2_(T/NT) < 2), and 2078 non-methylated piRNAs (log_2_(T/NT) < −2) show that *PIWIL1* transcripts were highly abundant in COLO 205 and medium-low expressed in SW-1417 cell lines, while *PIWIL2* and *PIWIL4* genes were expressed at low levels in 6 out of 8 cell lines and *PIWIL3* was undetectable in all cases with HCT 116 and RKO cells negative for all *PIWIL* transcripts.

To investigate the relationship between PIWILs and piRNAs in cancer, we focused on CRC cell lines as model systems. To this end, we evaluated expression of *PIWIL* genes in the European Genome-Phenome Archive (EGA) [37], a database containing RNA-Seq data from a collection of 53 cell lines isolated from CRCs. Interestingly, the molecular heterogeneity of *PIWIL* expression observed in CRC biopsies was well recapitulated in CRC cell lines (Figure S2). According to gene expression data, we selected 8 CRC cell lines for further study. Real-time qRT-PCR was used to evaluate the expression level of all *PIWIL* genes in the 8 cell lines (Figure 2A). Results show that *PIWIL1* transcripts were highly abundant in COLO 205 and medium-low expressed in SW-1417 cells, while *PIWIL2* and *PIWIL4* were expressed at low levels in 6 out of 8 cell lines, and *PIWIL3* was undetectable in all cases, with HCT 116 and RKO cells negative for all *PIWIL* transcripts.

In line with CRC data from TCGA, we observed a demethylation of the CpG island in the *PIWIL1* promoter region in the two cell lines expressing the gene, SW-1417 and COLO 205 (Figure 2B, Table S1B). Loss of DNA methylation was also proportional to the level of expression of the gene; a direct relationship between the two data suggesting the existence of a common epigenetic mechanism responsible for *PIWIL1* re-expression in both CRC tissues and cell lines. WB analysis on total protein lysates from the selected cell lines confirmed the presence of PIWIL1 in SW-1417 and COLO 205 (Figure 2C).

Our attention was then focused on piRNAs, and small RNA-Seq was applied to evaluate the presence and abundance of these small non-coding RNAs in CRC cell lines. As a control for piRNA identification, human testis RNA was sequenced in parallel. Since piRNAs map to thousands of genomic regions, reads corresponding to miRNAs, rRNA-derived small RNAs, tRNA-derived small RNAs, and small non-coding RNAs from the Ensembl and Rfam databases were filtered out before piRNA annotation to avoid misidentifications (Table S3).

Nevertheless, we identified a high number of piRNAs (~6000, with expression value > 0.1 count per million, CPM) in all colon cell lines and ~15,000 in the germline (Table S4). Surprisingly, we found that piRNAs are transcribed in colon cancer cells independently from *PIWIL* expression, as a comparable number of piRNAs were identified in all cell lines analyzed. Previous studies reported the presence of low levels of piRNAs transcribed outside the germline, in somatic tissues [9]. We found that the piRNA population identified is expressed at relatively low levels also in CRC cells, where it represents a small percentage of all reads assigned to small RNAs, <5% compared to ~17% in human testis (Figure S3, Figure S4, Table S3). The lack of correlation between *PIWIL* expression and piRNA levels suggests that basal piRNA transcription may occur in CRC cells—and possibly in all tumors—independently of the presence and abundance of PIWIL proteins. As a consequence, small amounts of piRNAs are always detectable, with their relative number and abundance being linked to the cell type, rather than to the presence/absence of PIWIL proteins. In fact, specific piRNA expression profiles can be observed in different cell lines, as shown by a hierarchical clustering analysis (single-linkage, Pearson distance) for 2500 piRNAs (>1.55 CPM) sorted by their abundance in the COLO 205 cell line (Figure S5, [9]). Based on the assumption that piRNAs are engaged in the PIWI-induced silencing complex (piRISC) to regulate gene expression, we decided to focus on the PIWIL1–piRNA complex and analyzed it in detail in COLO 205 cells, where the highest amount of PIWIL1 was detected in the absence of other PIWIL proteins (see below).

### 3.3. Characterization of PIWI–piRNA Pathway in COLO 205

Since piRNA maturation from precursors, their interaction with PIWIL proteins, and consequent downstream activities are mediated by a number of cofactors, identified and thoroughly studied in the germline and conserved across a wide range of animals [5], the expression of key components of the PIWI–piRNA pathway was evaluated in COLO 205 cells by RNA-Seq.

Results summarized in Figure 2D and Table S5 show the expression of several human homologs of piRNA-related genes previously described in the animal germline cells, including several factors involved in primary piRNA biogenesis and nuclear exporting. These include, together with factors involved in piRNA loading in PIWIL proteins and intermediate processing, DDX6, an RNA helicase, and the Tudor domain-containing protein TDRD7, belonging to the piRNA processing machinery in the germline, where it has been shown in multiprotein structures of the perinucleus called nuage [38]. On the other hand, it was not possible to detect expression of any factor involved in the secondary piRNA biogenesis pathway.

These results support a growing body of evidence indicating that genes of this pathway are expressed in different types of cancer, so this pathways is active and could play a key role in cancer cells. Recently, a *PIWIL1* knock-out experiment in COLO 205 cells showed a mild global effect on gene expression [39]. Differential expression analysis of these data accompanied by pathway analysis reveals, however, that several cellular processes and signaling pathways are significantly affected by PIWIL1 deficiency in these cells (Table S6).

It is noteworthy that we also noticed, among others, the presence in COLO 205 of HENMT1, the methyltransferase responsible for 3′-end 2′-*O*-methylation of piRNAs, a typical feature of these small RNAs shared among all organisms [40]. Based on this result, the 3′-methylation status of piRNAs was investigated in triplicate by sodium periodate/β-elimination treatment of total RNA extracted from COLO 205, followed by RNA sequencing (Table S3). As an internal control, to monitor the efficiency of the treatment, a pool of 4 synthetic molecules, two carrying the 3′-end methyl group characteristic of piRNAs and two with a free 3′-OH, as miRNAs and other small RNAs were spiked-in in each sample before processing. RNAs lacking the 3′-end methyl group were cleaved at the terminal ribose, preventing their cloning during library preparation and, therefore, detection by subsequent sequencing, while methylated molecules were incorporated and sequenced. Results showed that depletion efficiency for non-methylated RNA molecules, calculated from the spike-in abundance, was close to 98% (Figure S6). Sodium periodate oxidation had a strong impact on library composition, dramatically depleting non-methylated molecules such as miRNAs (from 80% to 3%), while a 3-fold increase was observed in piRNA reads (from 3% to 9%). As a control, the experiment was performed in parallel on a human testis RNA sample, where piRNA reads underwent to a 2.15-fold increase after the treatment (from 17.10% to 36.82%, Figure S4). Specifically, most of COLO 205 piRNAs (54.6%) were still detectable after the treatment, but the relative abundance and composition of the piRNA population in the library was strongly impacted. In fact, several abundant piRNAs were depleted by the treatment, while many less abundant molecules were efficiently sequenced (Figure S7). This last result can be explained considering both variable methylation levels of individual piRNAs and different library incorporation efficiency for 3′-end methylated and non-methylated molecules, since non-methylated RNAs are cloned with high efficiency, contrary to methylated ones that are, instead, incorporated with much lower efficiency [41]. On the other hand, methylated piRNAs are better detected after the treatment, due to the massive removal of non-methylated molecules and consequent reduction of competition during the enzymatic steps (Figure S7). Sequencing data showed that many piRNAs present in this cell line are methylated, including both completely and partly methylated ones. According to the ratio between piRNA detection levels before (NT) and after (T) treatment, we grouped those expressed in COLO 205 cells in three categories: 767 highly methylated (log_2_(T/NT) > 2), 1514 partially methylated (−2 < log_2_(T/NT) < 2), and 2078 non-methylated (log_2_(T/NT) < −2) (Figure 2F and Table S7).

Other categories were enriched by sodium periodate/β-elimination treatment, including tRNAs (from 7% to 16%) and those listed as Ensembl RNAs (snRNA, snoRNA, lincRNA, lncRNA, etc.), suggesting the presence of methylated species also in one or more of these classes of sncRNAs (Figure 2E), as reviewed by Dimitrova et al. [42]. It is also interesting to note that a strong enrichment upon treatment (from 2% to 30%, 468 methylated (log_2_(T/NT) > 2) and 225 partially methylated (−2 < log_2_(T/NT) < 2) molecules, Figure 2E and Table S7) was found also for unannotated reads, a category that comprises RNAs detectable by sequencing but not yet listed in current databases, some of which could represent still-uncharacterized piRNAs.

### 3.4. PIWIL1 Localizes in a Nuage-Like Structure of COLO 205 Cells

**Figure 3:**
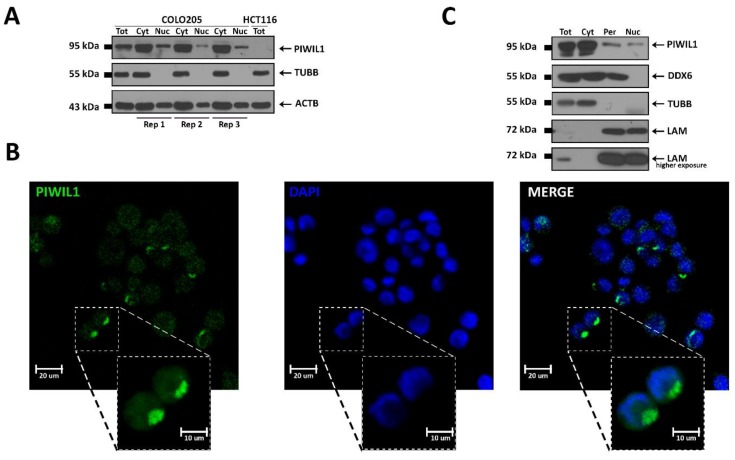
Characterization of PIWIL1 subcellular distribution. (**A**) Cell fractionation performed in triplicate in COLO 205 cells revealed the presence of PIWIL1 in both cytoplasmic and nuclear compartments; as a comparison, total protein lysate from COLO 205 and from HCT 116 (negative control) were run on the same gel; β-actin (ACTB) was used as a loading control, while β-tubulin (TUBB) was used to verify the absence of cross-contamination between cytosolic and nuclear fractions. (**B**) Localization of PIWIL1 protein (green) in COLO 205 cells by immunocytochemical analysis revealed its presence in both compartments, nucleus and cytosol, with especially bright staining in a discrete region located in proximity of the nucleus (blue, DAPI staining). (**C**) Cytosol/perinucleus/nucleus fractionation showed the presence of PIWIL1 in the perinuclear region of COLO 205 cells; in the same fraction, DDX6 protein, a marker for the human germline nuage compartment, was detected; TUBB was used to verify the absence of cytoplasmic contamination of perinuclear and nuclear fractions and β-lamin (LAM) as a control for perinuclear and nuclear extraction. As a comparison, total protein lysate from COLO 205 was loaded on the same gel.

Since PIWIL proteins can perform both cytoplasmic and nuclear functions [43], we investigated the subcellular localization of PIWIL1 in COLO 205 (Figure 3), SW1417, and *PIWIL1*-transfected HCT 116 cells (Figure S8). Fractionated protein extraction revealed that most of the protein concentrates in the cytoplasm (Figure 3A and Figure S8A–C), a result confirmed by immunocytochemistry, which revealed how a sizeable fraction of the protein is concentrated in a discrete cytoplasmic region leaning against the nucleus (Figure 3B and Figure S8D). This discrete region is more evident in COLO 205 cells, which express a high level of PIWIL1, compared to SW1417, where the protein is much less abundant (compare Figure 3B and Figure S8D). These results were also confirmed in PIWIL1-transfected HCT 116 cells where the IF signal, diffused throughout the cytoplasm, is clearly evident around the nucleus (Figure S8D).

In germline, PIWIL proteins and their interactors localize in proximity of the nucleus in a structure called nuage, where piRNA precursor transcripts are exported from the nucleus and biogenesis is thought to occur. To test the possibility that these somatic cells might contain a similar structure, we used a more stringent cell fractionation protocol [25] to isolate the perinuclear region of COLO 205, which was found to contain approximately 20% of the total protein content (Figure S9), and confirmed that ~10% cellular PIWIL1 is localized in the perinuclear region, ~4% in the nucleus, and the remainder in the cytosolic fraction (Figure 3C and Figure S9). Importantly, in the perinuclear fraction of COLO 205, we detected by WB also the presence of the nuage component DDX6 [44] (Figure 3C), which was also present in the cytoplasmic but not in the nuclear fraction. This result is of particular interest since it provides, for the first time, evidence that the perinuclear region of colon cancer cell lines contains a structure with similar localization and protein markers of the germline nuage compartment, the site of piRNA maturation and main actions in germline cells.

### 3.5. Identification of RNAs Associated with PIWIL1 in COLO 205 Cells

**Figure 4:**
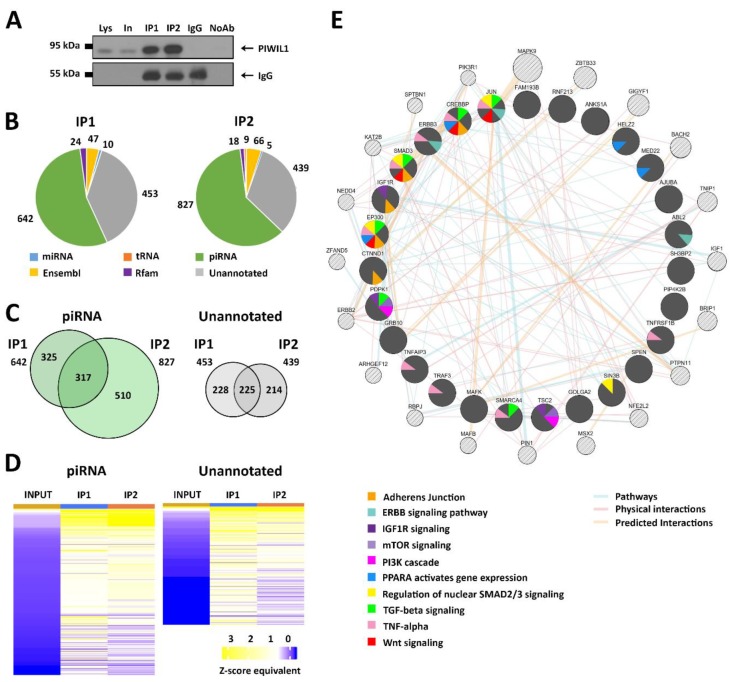
Identification of PIWIL1-bound RNAs. (**A**) RNA immunoprecipitation (RIP) was performed in the cytosolic fraction of COLO 205 cells with two different antibodies (IP1 and IP2); the presence of PIWIL1 protein was assessed by western blotting in the cytosolic lysate (Lys), Input (In), IP1, and IP2; PIWIL1 was absent in negative controls (IgG and NoAb); IgG were used as a control for IP loading. (**B**) Pie chart representing the number of enriched molecules per category in both IP1 and IP2 samples; (**C**) Venn diagram summarizing the number of piRNAs (left) and unannotated molecules (right) identified with both antibodies; (**D**) heatmaps representing Z-score expression values for 317 piRNAs (on the left) and 225 unannotated RNAs (on the right) identified in PIWIL1 RIP-Seq with two different antibodies (IP1 and IP2), compared to the input RNA. (**E**) Network analysis and functional enrichment for 106 validated interactions revealed a group of 27 master-regulator genes targeted by the PIWIL1–piRNA complex and 20 correlated genes strongly related with molecular pathways crucial for CRC pathogenesis. Relations between genes (173 links, Table S9E) and association to pathways are represented with a color code. Abbreviations: ERBB, avian erythroblastosis oncogene B; IGF1R, insulin like growth factor 1 receptor; mTOR, mechanistic target of rapamycin kinase; PI3K, phosphoinositide 3-kinase; PPARA, peroxisome proliferator activated receptor alpha; SMAD2/3, mothers against decapentaplegic homolog 2/3; TGF, transforming growth factor; TNF, tumor necrosis factor; Wnt, wingless/int-1.

To identify and characterize the PIWIL1–piRNA complexes present in the COLO 205 cell line, RNA immunoprecipitation (RIP) was performed with PIWIL1-specific antibodies (Abs) on total protein extracts from exponentially growing cultures, followed by library preparation and sequencing. PIWIL1-bound RNAs were identified by performing independent reactions with two Abs (IP1 and IP2), each raised against a different PIWIL1 epitope; as a negative control, the protocol was carried out in parallel with non-specific IgGs as well as without antibody addition, and the whole experiment was performed on biological triplicates. As shown in Figure 4A, PIWIL1 was efficiently immunopurified by both Abs (Figure 4A). After data filtering and normalization, RNA molecules enriched in either IP1 or IP2 immunoprecipitated samples with respect to the input lysates (log_2_ FC > 2, FDR < 0.01) were identified. To exclude non-specific interactions, RNAs that resulted in being enriched also in the “No Antibody” control samples with respect to the input lysates were excluded from the analysis (Table S8). The two antibodies provided very similar results, showing that more than 50% of the PIWIL1-interacting RNAs identified correspond to known piRNAs, while most of the remaining RNAs are unannotated, suggesting that they could also include unclassified somatic piRNAs from COLO 205 cells (Figure 4B,C). The enrichment factor, calculated for each piRNA in immunoprecipitated samples with respect to its concentration in the input lysates, ranged between 4 and 64, and a similar behavior was observed for unannotated RNAs (Figure 4D and Table S8). The other small RNAs found associated with PIWIL1 by RIP include some miRNAs and sncRNAs included in the Ensembl and Rfam databases, comprising some (in particular, snoRNAs) that are considered piRNA precursor transcripts [45,46]. In order to focus on the most reliable data, the results considered consisted only of molecules immunopurified with both antibodies, represented by 583 small RNA species associated with PIWIL1, including 317 piRNAs and 225 unannotated small RNAs, some of which could represent new somatic piRNAs (Figure 4D).

### 3.6. Identification of mRNAs Targeted by the PIWIL1–piRNA Complex

To investigate the biological function of the PIWIL1–piRNA complex in colon cancer, we performed a target prediction analysis for 317 PIWIL1-bound piRNAs. As a result, we identified 3288 putative target transcripts expressed in the COLO 205 cell line, as determined by RNA-Seq. Interestingly, the number of piRNA target sequence was greater in the 3′UTR of the RNA (2673) compared to the 5′UTR (499) or the Coding Sequences (CDS) region (524) (data not shown). To search for biological processes involving the PIWIL1–piRNA complex, we performed a pathway analysis based on the known functions encoded by the 3288 mRNAs. Results showed a significant enrichment in functional categories involved in the cell cycle, including gene expression, circadian clock, G2/M transition, and metabolism, some of which become dysregulated in the same cell line upon PIWIL1 deprivation (highlighted in bold in Table S9A). In addition, this functional analysis revealed several transcripts encoding key components of signal transduction cascades frequently found deregulated in CRC, including the TGF-β, Wnt, mTOR, and PI3K pathways. Interestingly, several other processes potentially regulated by the piRNA pathway emerged, the roles of which are still poorly understood in CRC; among these, it is worth mentioning the signaling pathway comprising of histone deacetylase (HDAC) class II molecules, TNF-alpha, and PPAR-alpha (Figure S10, Table S9A).

Based on the consideration that PIWIL1 is in direct contact with piRNAs and their target in the piRISC complex, we reasoned that the RIP-Seq protocol also allows the simultaneous isolation of piRNAs and their target RNAs. To verify the presence of genes enriched in the piRISC, we mapped the longest reads (46 to 75 nucleotides long) generated from all small RNA-Seq datasets to the Ensemble transcripts database (http://www.ensemble.org). Then, following the same approach used for PIWIL1-bound piRNA identification, we identified a significant physical interaction between the PIWIL1–piRNA complex and 106 protein-coding transcripts (Table S9B) through sequence complementarity with 90 different piRNAs (Table S9C). The specific fold-enrichment calculated between the input and PIWIL1 immunoprecipitated samples varied between 1.5 and 8.3 for IP1 and 4.8 for IP2 Abs (Table S9B). These transcripts are most likely piRNA targets, and, importantly, they have been identified in the context of the piRISC in the absence of any crosslinking step, thereby avoiding possible artifacts produced by this treatment. To further analyze the gene network directly engaged by PIWIL1–piRNA complexes in COLO 205 cells, starting from the 106 experimentally validated target transcripts, we built a functional interaction network based on a combination of various functional descriptors shared between the partners, such as physical and predicted interactions and functional pathways, using the GeneMANIA platform (http://www.genemania.org) [47]. Results show 27/106 genes (dark gray in Figure 4F), as well as 20 additional related genes (light gray) predicted by GeneMANIA, mapped to an interconnected network showing 173 interactions (Table S9D and S9E). In this context, the most significantly affected functional pathways identified include, among others, TNF-alpha, SMAD2/3, TGF-beta, and Wnt signaling (Figure 4F), suggesting that specific PIWIL1–piRNA–mRNA interactions may be controlling essential cellular functions in CRC cells by post-transcriptional gene regulation mechanisms similar to those identified for PIWIL and piRNAs in germline cells.

## 4. Discussion

An increasing number of studies have demonstrated that germline PIWIL proteins and associated piRNAs are dysregulated in cancers and can be used as diagnostic and prognostic biomarkers [15,48,49]. However, the biological role of the piRNA pathway in pathological conditions is still undefined, principally due to the lack of studies focused on the identification of the molecular targets of the PIWIL–piRNA complex in cancer cells. Interestingly, *PIWIL* genes have been included in the list of cancer testis antigens (CTAs), germline genes whose expression is re-activated in cancer, which are generating great interest in the scientific community as possible druggable targets. In particular, *PIWIL1* has been recently listed among the extremely high-expressed cancer testis genes (EECTGs) and are considered as a potential epi-drivers in tumors [33].

Based on a meta-analysis of TCGA and GTEx data, we found that *PIWIL1* presents unique features among *PIWILs*, as *PIWIL2* and *PIWIL4* are frequently deregulated in cancers but are also expressed in several normal tissues, while *PIWIL1* is almost absent in non-transformed tissues. Furthermore, ectopic expression of this germline-specific gene was almost exclusively detected in CRC, where it is significantly upregulated in 11% of tumors, with respect to normal samples. Based on DNA methylation data, the expression of *PIWIL1* in human colorectal cancers and cell lines is directly associated with the hypomethylation of 6 CpG sites in its promoter region, an event frequently linked to CTA re-expression in pathological conditions. These results allow us to hypothesize that in normal human colon cells, the majority of CpGs in the *PIWIL1* promoter are methylated, leading to gene silencing, whereas loss of DNA methylation, which frequently occurs during malignant transformation for delays in maintenance of methylation during accelerated cell division, could in this case drive abnormal *PIWIL1* expression in a subgroup of CRCs, as also observed in lung adenocarcinomas [34].

During the last few years, piRNA expression profiles have been investigated in several human tumors [10,48], but the association of these small RNAs with PIWILs and their involvement in CRC are still poorly investigated. Interestingly, by directly comparing *PIWIL* and piRNA expression levels, we noticed that the presence/absence of *PIWIL* gene expression is not directly related to a specific piRNA expression pattern. Indeed, small RNA-Seq analyses in 8 CRC cell lines revealed that a low percentage of piRNAs (<7%) is detectable in all cases, independently from the expression patterns of *PIWIL* genes, a result supported by gene knock-down experiments in somatic tissues that demonstrated how *PIWIL1* depletion does not significantly affect piRNA levels [50,51]. It is thus possible to assume that PIWIL1 is not required for piRNA biosynthesis and/or maturation in CRC and, more generally, in somatic tissues. On the other hand, this Argonaute protein is associated with a subset of piRNAs (317 molecules) and other unannotated small RNAs, as demonstrated by PIWIL1 RIP-Seq in CRC cells (Figure 4C). This evidence, combined with the detection of several members of the piRNA biosynthetic and effector pathway in these same cells (Figure 2D) and the finding that a large fraction of the expressed piRNAs (>50%, Figure 2F) are represented by biologically active methylated molecules, indicates a role for these regulatory RNAs in CRC. Furthermore, the fact that piRNAs are produced in CRC by primary processing and not amplified through the secondary amplification pathway explains their relatively low abundance in these cells, compared to germline ones, in agreement with what was previously demonstrated in other somatic cells [52,53,54].

Across the animal kingdom, PIWIL proteins and associated piRNAs have been found in both nuclei and cytoplasm, suggesting that the piRNA pathway may function in both these cellular compartments [55] and, therefore, may exert multiple roles. Using cell fractionation and immunocytochemistry, we found that PIWIL1 is primarily localized in the cytoplasm, but, surprisingly, we also observed the existence of a nuage-like structure in the perinuclear space, where the protein is much more concentrated than in the rest of the cell, in analogy with what occurs in the germline nuage compartment, where piRNAs are exported and loaded onto PIWIL proteins. Importantly, in the perinuclear fraction of COLO 205 cells, we also detected DDX6, an RNA helicase responsible for piRNAs biogenesis and marker of the germline nuage [44]. Since impairment of the piRNA pathway in animals led to disruption of the nuage compartment [56], together with the evidence that these subcellular structures may serve to increase the local concentration of specific proteins of the pathway and of piRNAs, this compartmentalization observed for PIWIL1 represents a strong indication that the piRNA pathway is functional in these cells. This is further supported by experimental identification of a physical interaction between PIWIL1 and 317 piRNAs, as well as 225 unannotated small RNAs that might represent new somatic piRNAs in CRC cells. It is noteworthy that Yin et al. [57] have recently identified 35 piRNAs deregulated in CRC biopsies, with respect to paired normal mucosa, and found a correlation between the expression of some of these RNAs and tumor differentiation and lymph node metastasis. Interestingly, 4 of these piRNAs were also found in our dataset: piR-hsa-28319 (hsa_piR_020450 in piRNABank), identified among PIWIL1/bound molecules in the RIP-Seq experiment, piR-26039 (hsa_piR_018780) and piR-17560 (hsa_piR_012753), enriched in PIWIL1 IP but classified as snoRNA-derived sncRNA (Ensembl molecules), and piR-28317 (has-piR-23317), identified by small RNA-Seq of COLO 205 but not found associated with PIWIL1 by RIP. This evidence supports the results reported here and further suggests that this pathway may indeed exert important functional roles in CRC.

In recent years, an increasing number of post-transcriptional regulation mechanisms for primary piRNAs, beyond transposon silencing, have been described as piRNAs that can guide PIWI proteins to destabilize specific mRNA targets via RNA–RNA interactions by a miRNA-like mechanism [58], but they can also stabilize mRNAs [59]. Interestingly, piRNA target prediction and RNA immunoprecipitation confirmed the interaction between the PIWIL1–piRNA complex and at least 106 COLO 205 transcripts. In this context, interaction network analysis identified that 27 such transcripts are encoded by genes involved in key pathways that are frequently found deregulated during CRC carcinogenesis and cancer progression. Among these, 8 tumor-suppressor genes are worth mentioning (EP300, CREBBP, CTNND1, SMAD3, SMARCA4, SPEN, TNFAIP3, and TSC2), as well as genes that control normal cell proliferation and differentiation, such as IGF1R, JUN, and ERBB3, to suggest the interesting possibility that the PIWIL1–piRNA machinery may participate in tumorigenesis by directly regulating the activity of these genes. Indeed, PIWIL1 expression in CRC has recently been correlated with tumor differentiation, infiltration, lymph node invasion, and metastasis [17] and has been tightly associated with *BRAF* gene mutation (V600E) and poor prognosis [60]. It is worth noting that Herr et al. have observed that the treatment of COLO 205 cells with BRAF inhibitors induces a strong downregulation of PIWIL1, suggesting that the activation of PIWIL1 in this cell line may be responsible for their less differentiated and a stem-like signature and, therefore, for their metastatic behavior, and may contribute to the aggressive phenotype of BRAF-mutated tumors [61]. The PIWIL/piRNA-induced regulation of gene expression is a complex mechanism, finely regulated in multiple points, with functions performed in both nuclear and cytoplasmic compartments [49]. Here we focused our attention on the molecular interaction between PIWIL1/piRNA complexes and target mRNAs in the cytosolic fraction of COLO 205; however, it is interesting to note that there is also evidence for a PIWIL1-mediated epigenetic regulation of gene expression in CRC [62]. Moreover, Siddiqi et al. [63] demonstrated, in human sarcoma, that HIWI (PIWIL1) mediated tumorigenesis is associated with global DNA-hypermethylation and silencing of tumor suppressor genes; it is interesting to note, among them, is present SMAD3, also identified here among the molecular target of PIWIL1/piRNA complexes. Furthermore CREBBP, EP300, ERBB3, IGF1R, JUN, SMAD3, SMARCA4 and TSC2 genes, identified here as targets of PIWIL1/piRNA complexes, were found differentially expressed between consensus molecular subtype (CMS) groups in primary CRCs and metastases [64], thus suggesting the involvement of the PIWIL1–piRNA system as an regulator of the complex genetic network controlling early as well as advanced stages of CRC progression.

## 5. Conclusions

The present study confirmed recent findings of aberrant PIWIL proteins and piRNA expression in cancer but provides, for the first time, a molecular view of the involvement of these small regulatory RNAs and their pathway in CRC. We demonstrated that among *PIWIL* genes, *PIWIL1* presents some unique features, such as germline specificity and ectopic activation in CRC due to a loss of DNA methylation in the promoter region of the gene, and all peculiar features of CTAs, which are a promising target for the development of cancer-specific immunotherapies. CRC piRNAs present the molecular features required by these molecules to be functionally active, such as the methyl group at their 3′-end. Importantly, this study shows, for the first time in cancer cells, the presence of a nuage-like structure, proposed to be central for recruitment of functional components of the PIWI–piRNA pathway and selection and processing of target mRNAs. Finally, we identified a PIWIL1-associated piRNA population involved in gene regulation events that may control basic features of colon cancer, providing in this way, a useful resource for further functional studies to decipher the role and importance of piRNA-mediated gene regulatory events in view of identifying new biomarkers and potential therapeutic targets.

## Figures and Tables

**Table 1:** Real-time qRT-PCR primers.

Gene	Forward (5′-3′)	Reverse (5′-3′)
***PIWIL1***	ACGAAGTGCCACAGTTTTTGG	AGTCTTCCTCCAGACTGAGC
***PIWIL2***	GCCTGGGTTGAACTAAAGGA	CCATGATGATGCAAACAACC
***PIWIL3***	TCAGATGGCAGCAAAATCAC	ACGTTGTGTACCCGTTAGGC
***PIWIL4***	ATGGCACCGAGATCACCTAT	GCTGAGCCTCACTGTTGTCA

## Supplementary Materials

The following are available online at https://www.mdpi.com/2073-4409/8/11/1390/s1. Figure S1: Expression of *PIWIL2*, *PIWIL3*, and *PIWIL4* genes in cancer tissues; Table S1: Methylation and gene expression data of TCGA colon cancer tissues (S1A) and CRC cell lines (S1B); Figure S2: Molecular heterogeneity of *PIWIL* genes in tumors is maintained in CRC cell lines; Table S2: PIWIL1 CRISPR–Cas9 loss-of-function screening, Table S3: Mapping statistics of samples analyzed by small RNA sequencing; Figure S3: Small RNA profiling of CRC cell lines; Figure S4: Effect of the sodium periodate (NaIO_4_)/β-elimination treatment in human testis RNA; Table S4: piRNA expression in human colon cancer cell lines and testis; Figure S5: Relative abundance of CRC piRNAs compared to the germline; Table S5: Expression of piRNA pathway genes in COLO 205; Figure S6: Efficiency of the sodium periodate (NaIO_4_)/β-elimination treatment; Figure S7: Heatmaps showing the effect of the NaIO_4_ treatment on the piRNA population in COLO 205; Table S6: Pathway analysis of *PIWIL1* loss of function in COLO 205 cells; Table S7: Relative mean ratios and count per million (CPM) values of samples treated with sodium periodate/β-elimination in the COLO 205 cell line; Table S8: Enrichment values for RIP-Seq experiment; Figure S8: Characterization of PIWIL1 subcellular distribution; Figure S9: Evaluation of subcellular distribution of PIWIL1 in the COLO 205 cell line; Figure S10: Pathway analysis of putative piRNAs mRNA targets; Table S9: Analysis of PIWIL1–piRNAs target genes in COLO 205; Supplementary Methods.
