# Supplementary material for: Molecular and Functional Characterization of the Somatic PIWIL1/piRNA Pathway in Colorectal Cancer Cells
Source: Cells. 2019 Nov 5;8(11):1390. doi: 10.3390/cells8111390 (PMC6912267; doi:10.3390/cells8111390)
Supplement: Supplementary file 1 [file cells-08-01390-s001.zip › Sellitto et al Supplementary Materials.docx]

*Supplementary Materials*

Molecular and functional characterization of the somatic PIWIL1/piRNA pathway in colorectal cancer cells

**Assunta Sellitto, Konstantinos Geles, Ylenia D’Agostino, Marisa Conte, Elena Alexandrova, Domenico Rocco, Giovanni Nassa, Giorgio Giurato, Roberta Tarallo, Alessandro Weisz and Francesca Rizzo.**


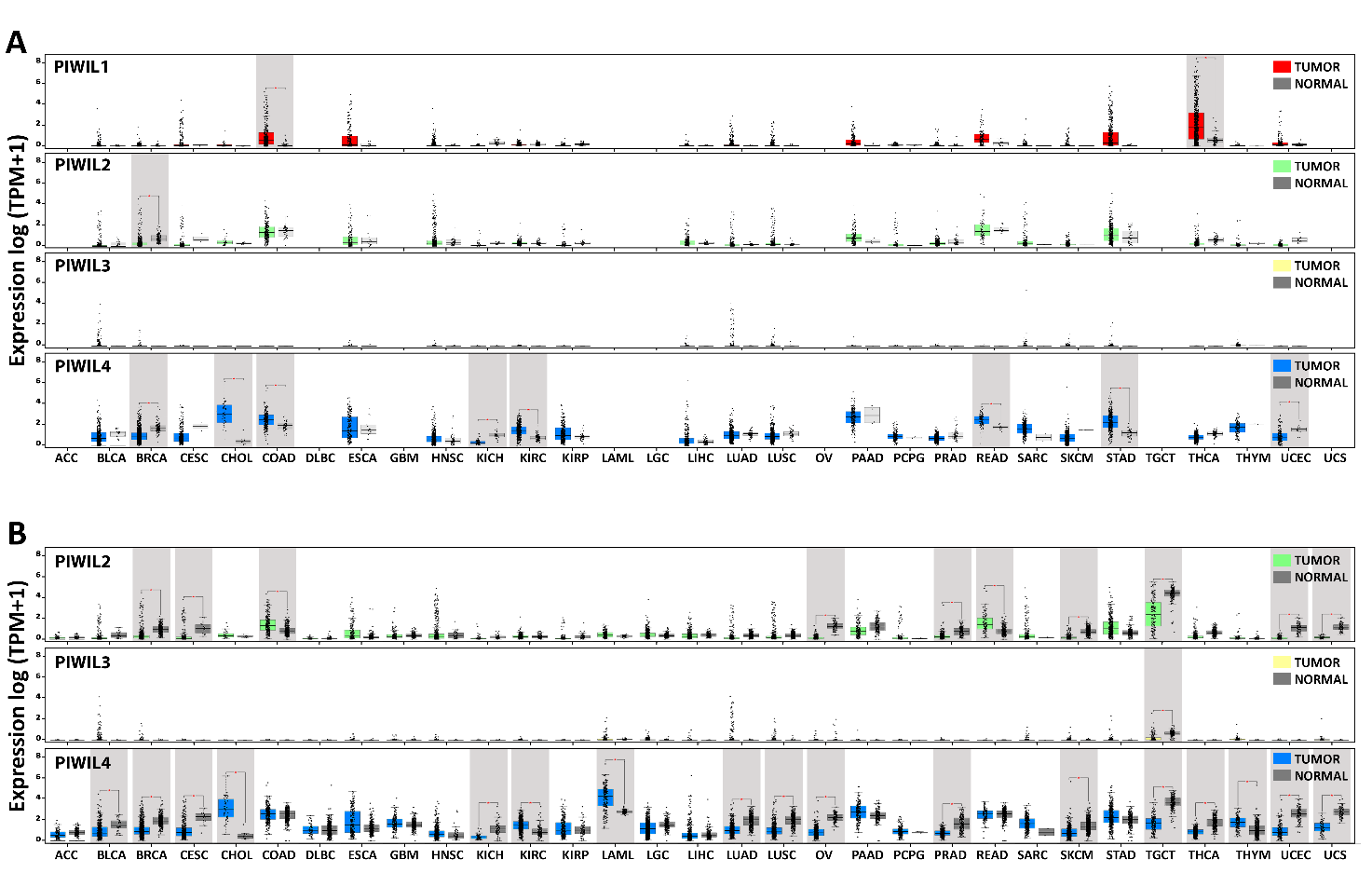


**Figure S1.** Expression of *PIWIL* genes in cancer tissues. Gene expression analysis of TCGA data for *PIWIL1*, *PIWIL2*, *PIWIL3* and *PIWIL4 genes* (A) and of GTEx/TCGA integrated data for *PIWIL2*, *PIWIL3* and *PIWIL4* genes (B) in tumoral and normal samples. Significantly up- or down-regulations in carcinomas with respect to the normal tissue (*p* value <0.05) are highlighted in grey; expression levels are displayed in log_2_-transformed (TPM (Transcripts Per Million) +1).


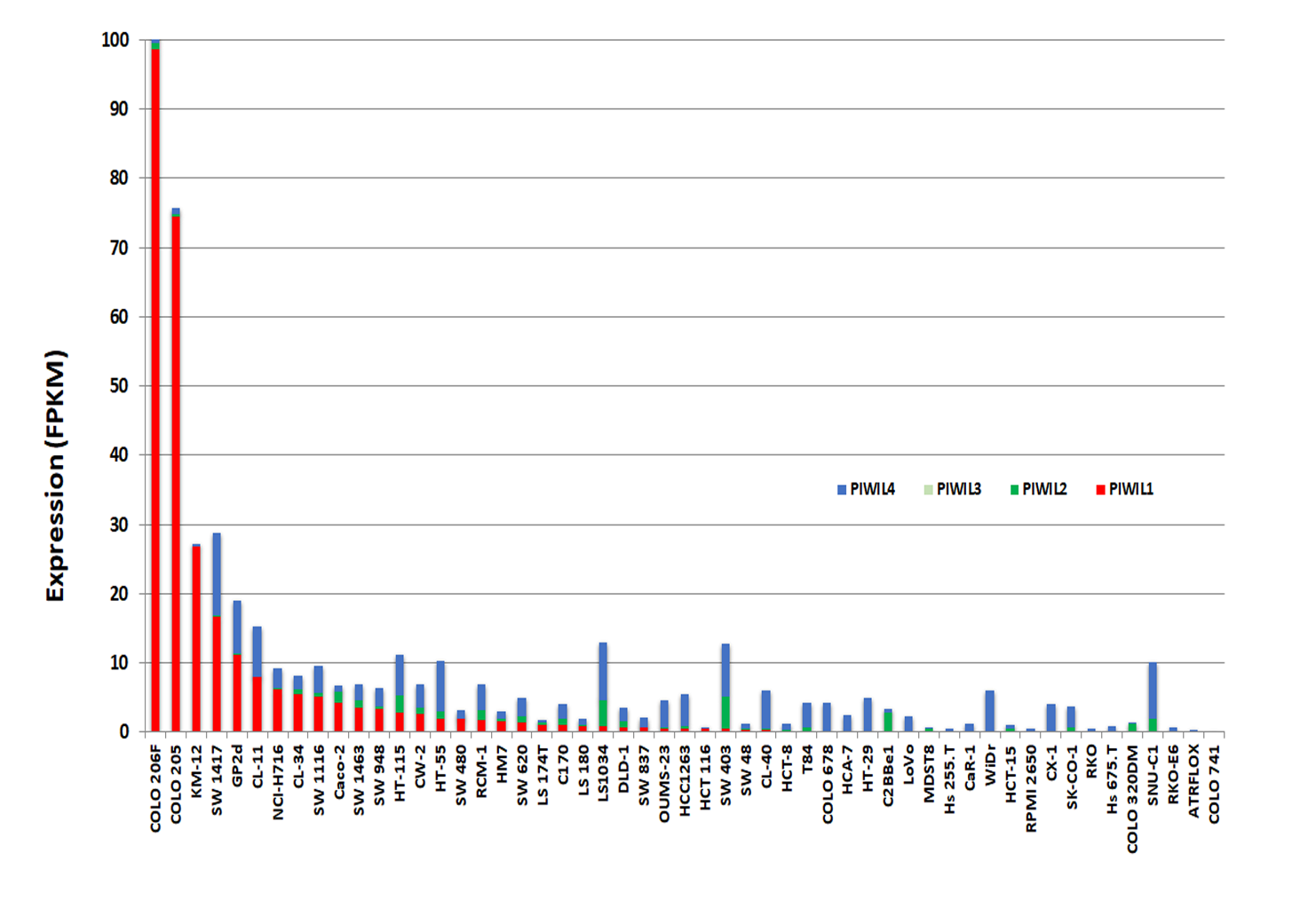


**Figure S2.** The molecular heterogeneity of *PIWIL* genes in tumors is maintained in CRC cell lines. Expression of *PIWIL* genes in 53 CRC cell lines listed in the EGA database (E-MTAB-2706); expression levels are displayed in FPKM (Fragment Per Kilobase Million).


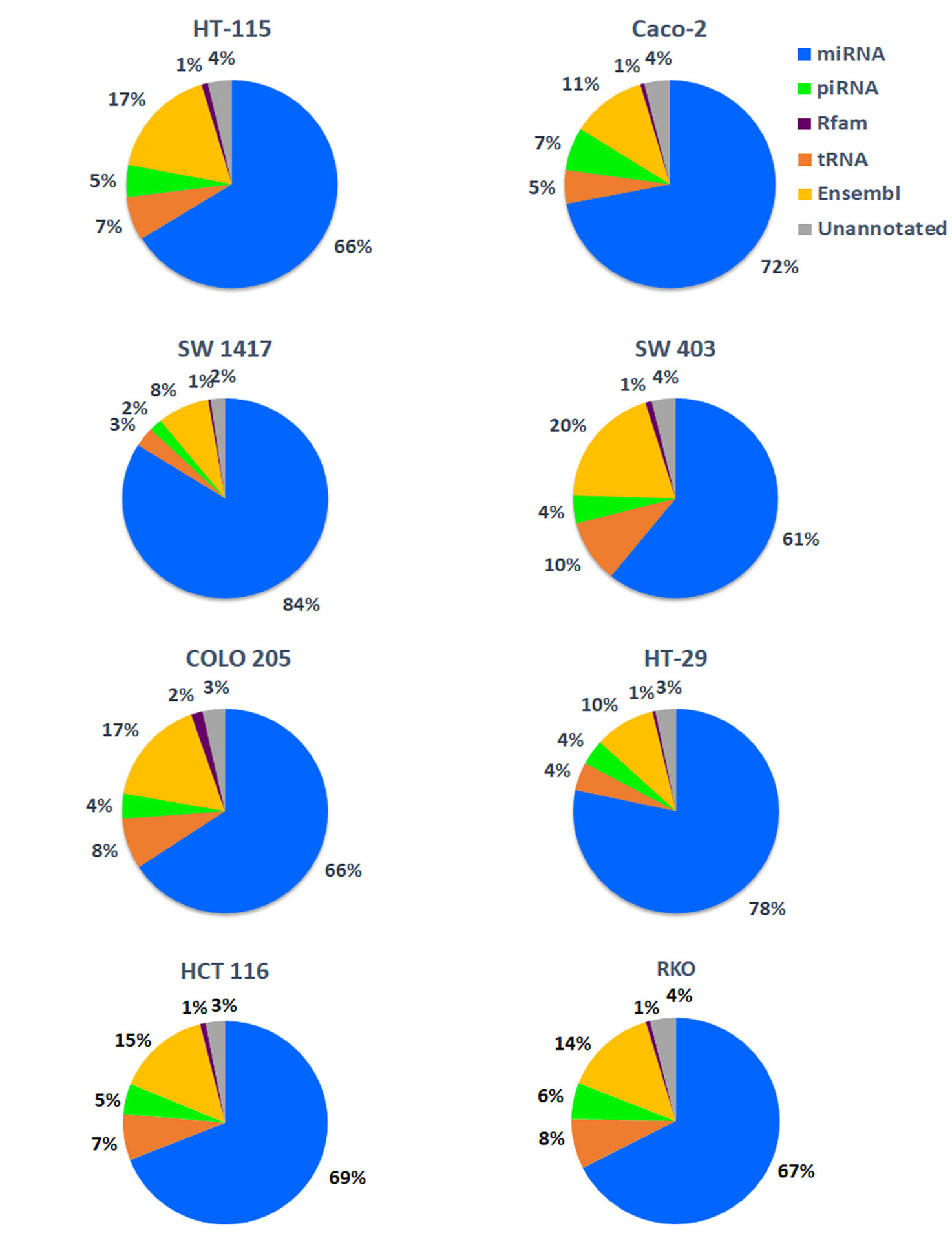


**Figure S3.** Small RNA profiling of CRC cell lines. Pie-charts representing the relative abundance of small RNA categories identified in CRC cell lines; small RNA-Seq analysis confirmed the presence of piRNAs in all cell lines.

**
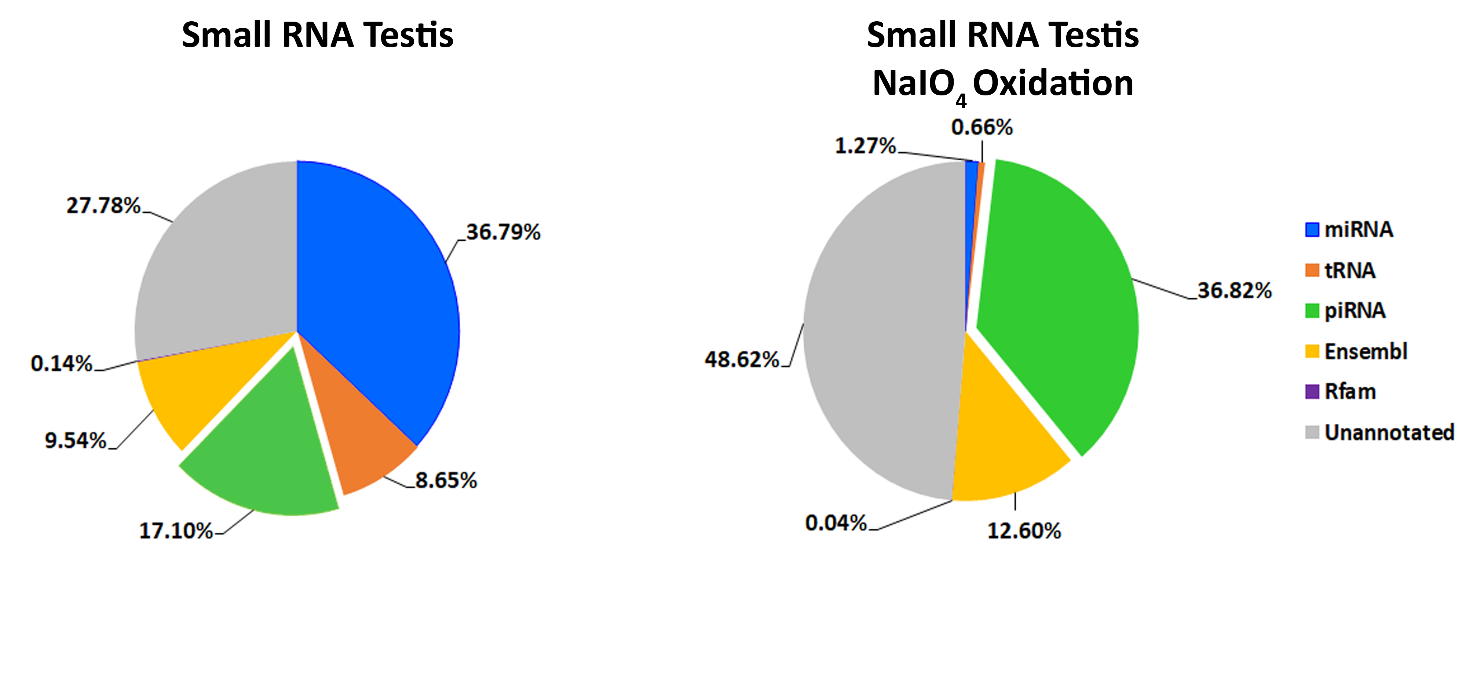
Figure S4.** Effect of the sodium periodate (NaIO_4_)/β-elimination treatment in the Human Testis RNA. Pie-charts representing the relative abundance of small RNA categories identified in the Human Testis RNA before (right) and after (left) the sodium periodate/β-elimination treatment; small RNA-Seq analysis confirmed the resistance of piRNAs to the treatment and the depletion of non-methylated molecules.

**
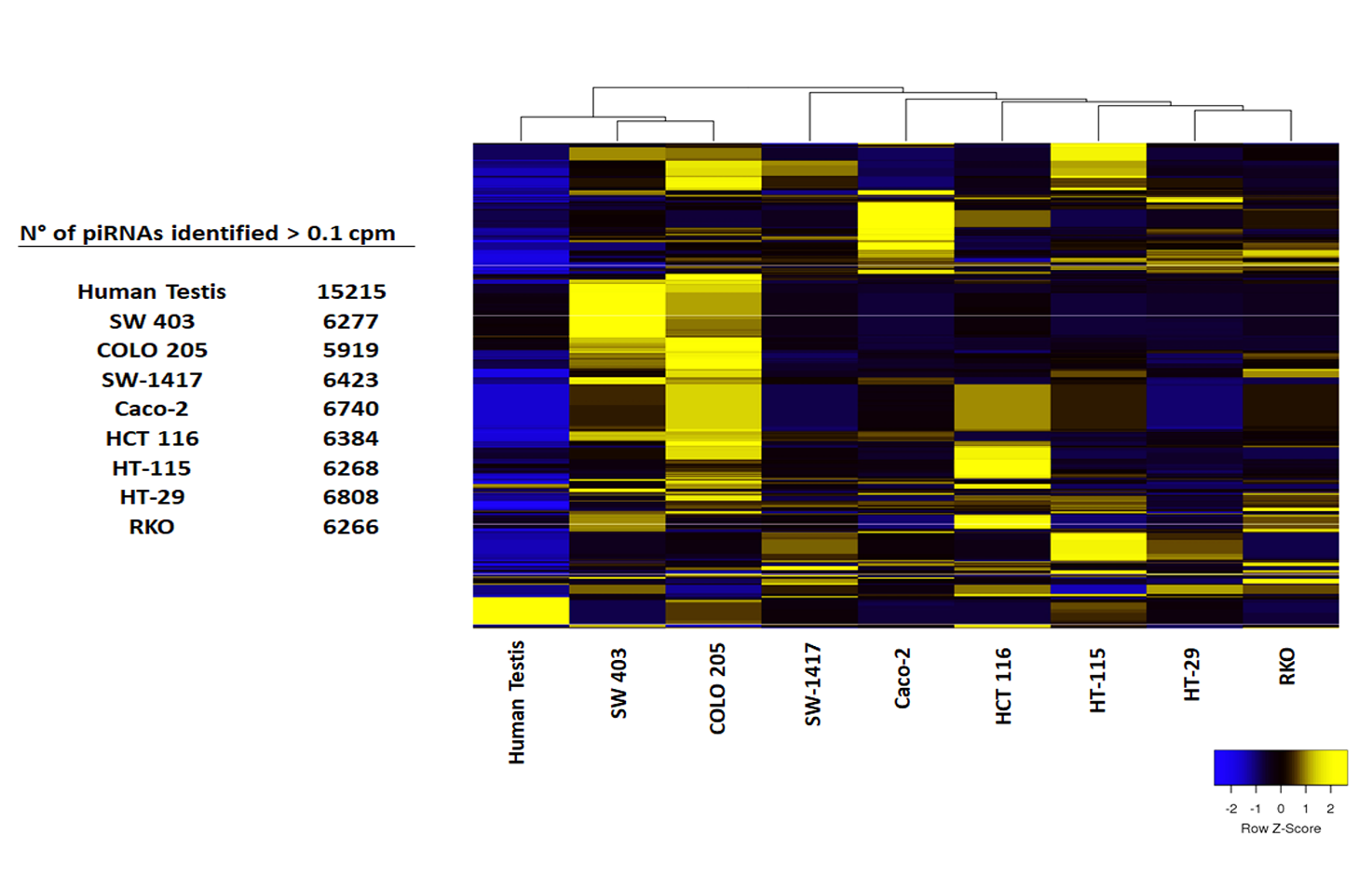
**

**Figure S5.** Relative abundance of CRC piRNAs compared to the germline’s one. Heatmap representing the expression levels of 2500 piRNAs in CRC cell lines compared to the Human Testis control. piRNAs were selected from the whole dataset according to their abundance in the COLO 205 cell line; Z-score expression values are displayed from the blue (low abundance) to the yellow (high abundance); the complete number of identified piRNAs in each cell line and in the Human Testis is indicated in the table on the right.

**
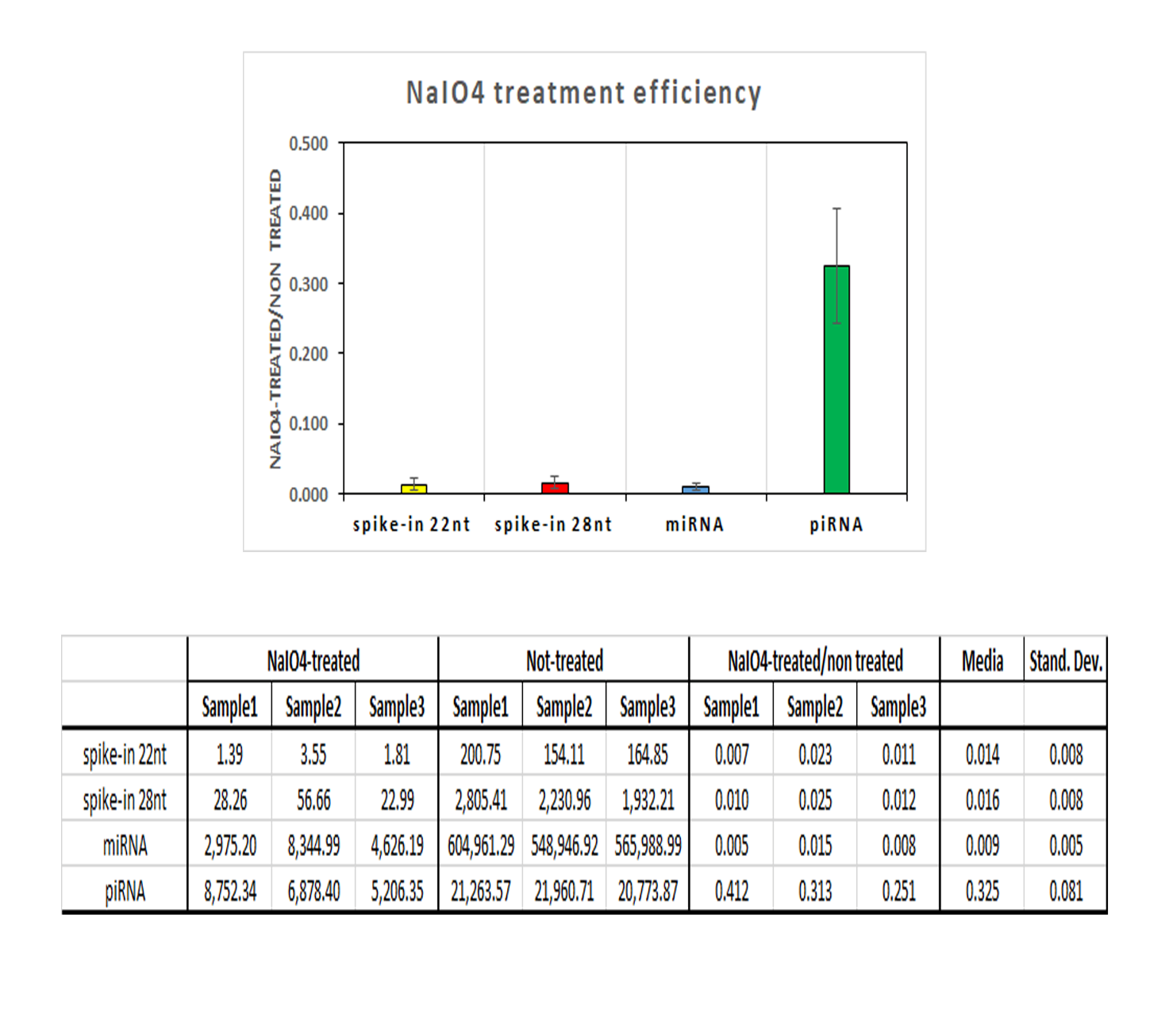
**

**Figure S6**. Efficiency of the sodium periodate (NaIO_4_)/β-elimination treatment. The treatment efficiency was evaluated comparing the normalized reads obtained after sequencing for two exogenous controls (synthetic non-methylated spike-in molecules) and for endogenous molecules (miRNAs and piRNAs) in treated and non-treated samples.


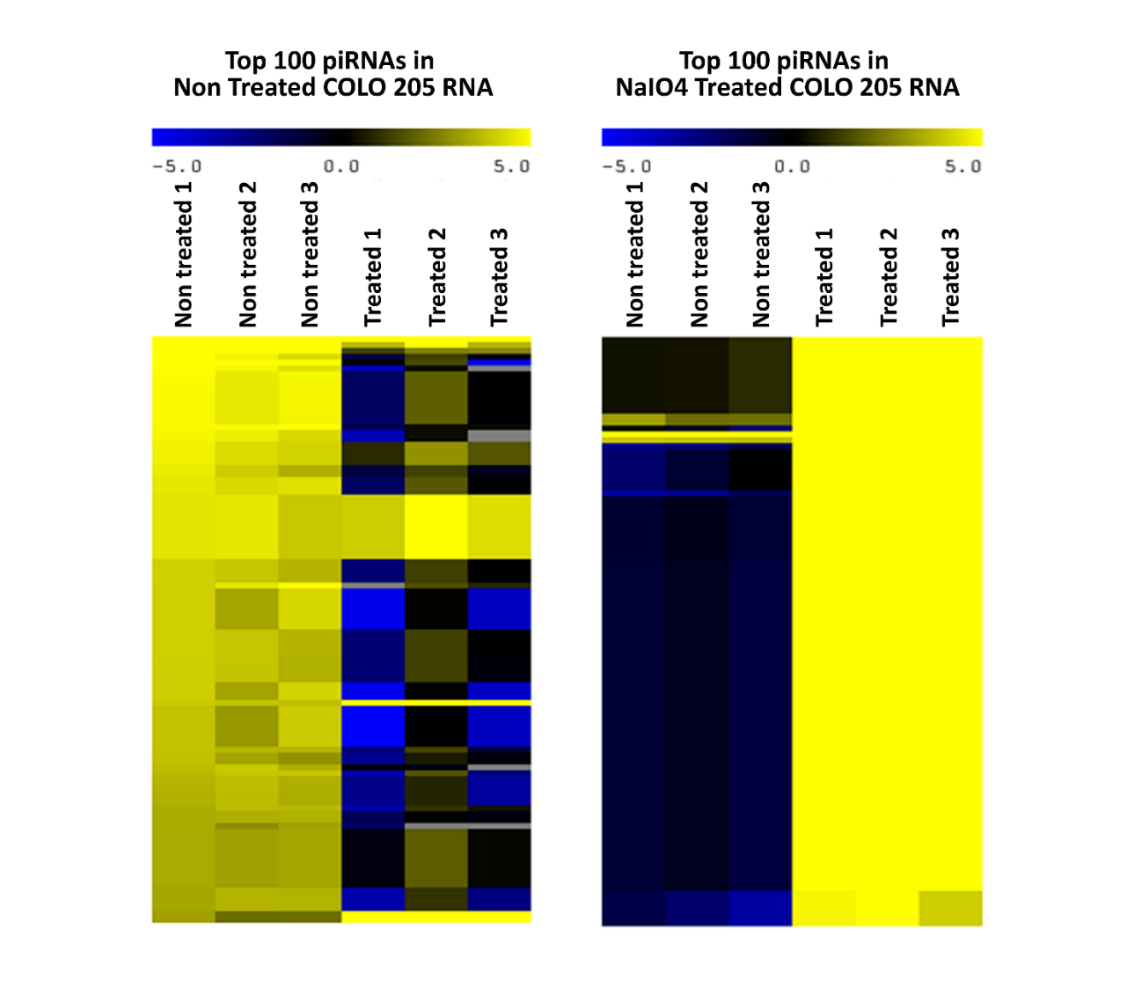


**Figure S7.** Heatmaps showing the effect of the NaIO_4_ treatment onto piRNAs population in COLO 205. Expression levels (log_2_ Count Per Million-CPM) are displayed for top 100 abundant piRNAs detected in non-treated total RNA (left) and in the NaIO_4_ treated total RNA (right).

**
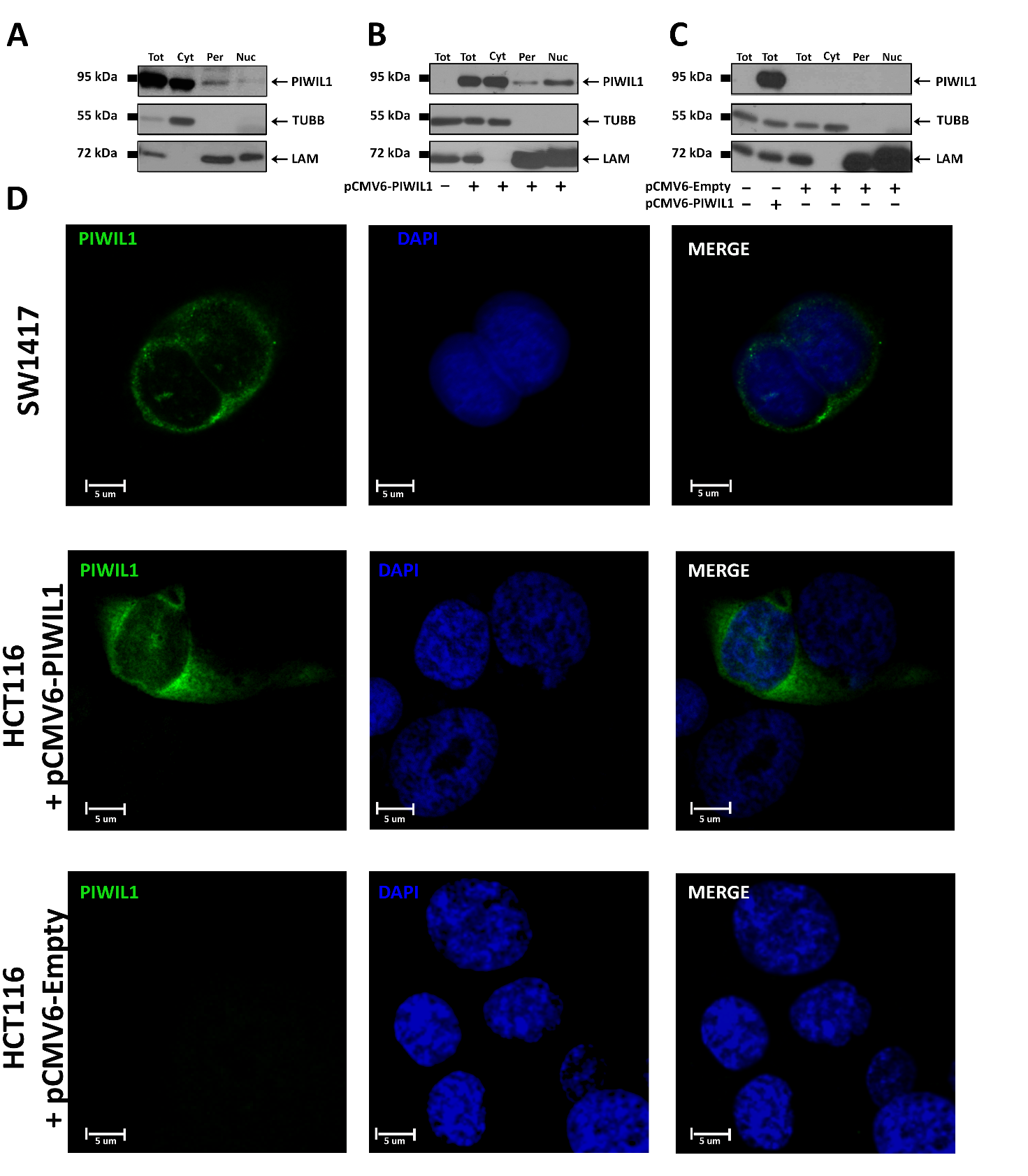
Figure S8. Characterization of PIWIL1 subcellular distribution. Western Blotting of Cytosol/Perinucleus/Nucleus fractionation showing the presence of PIWIL1 in the perinuclear region of SW1417 cells (A), HCT116 cells overexpressing PIWIL1 (B) and HCT116 cells transfected with empty vector (C); β-actin was used as a loading control, β-tubulin was used to verify the absence of cytoplasmic contamination of perinuclear and nuclear fractions. As a comparison, total protein lysate from was loaded on the same gel. (B) Localization of PIWIL1 protein (green) in SW1417 (top), HCT116 cells overexpressing PIWIL1 (center) and HCT116 cells transfected with empty vector (bottom). Nuclei are stained in blue (DAPI)**

**
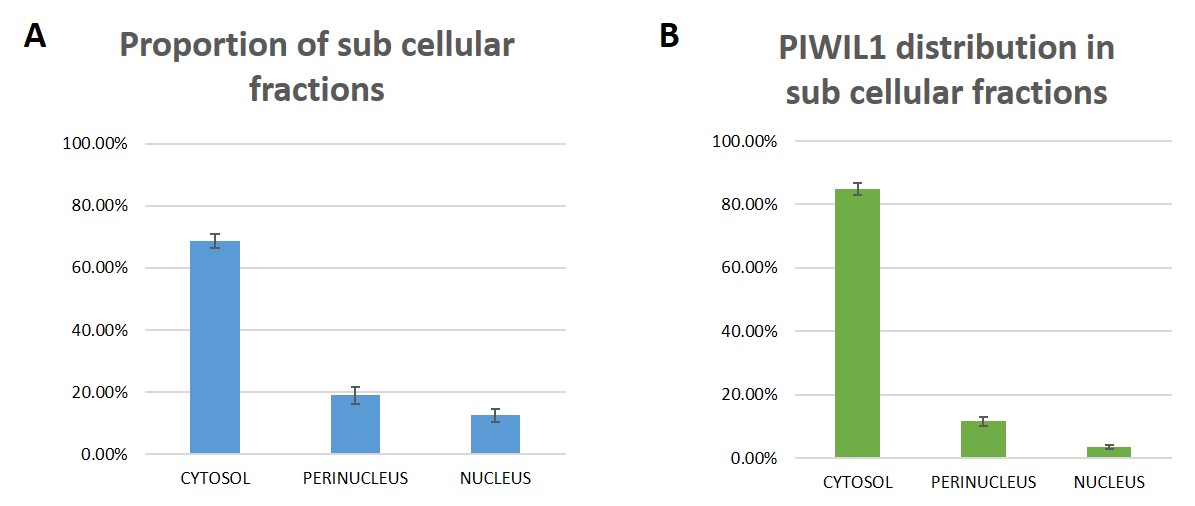
**

**Figure S9**. Evaluation of sub-cellular distribution of PIWIL1 in COLO 205 cell line. To evaluate the subcellular distribution of PIWIL1, three independent subcellular extraction from COLO 205 for the three subcellular fractions were performed: cytosolic fraction was found to contain approximately 69% of the total protein content, while perinuclear and nuclear fraction accounted for 19% and 12% respectively (A). The relative amount of PIWIL1 in each fraction was measured by band densitometry and corrected to reflect the same cell equivalent (B). PIWIL1 protein is primarily localized in the cytoplasm (~85%), although a discrete amount is present in the perinuclear (~12%) and nuclear (~4%) fractions.


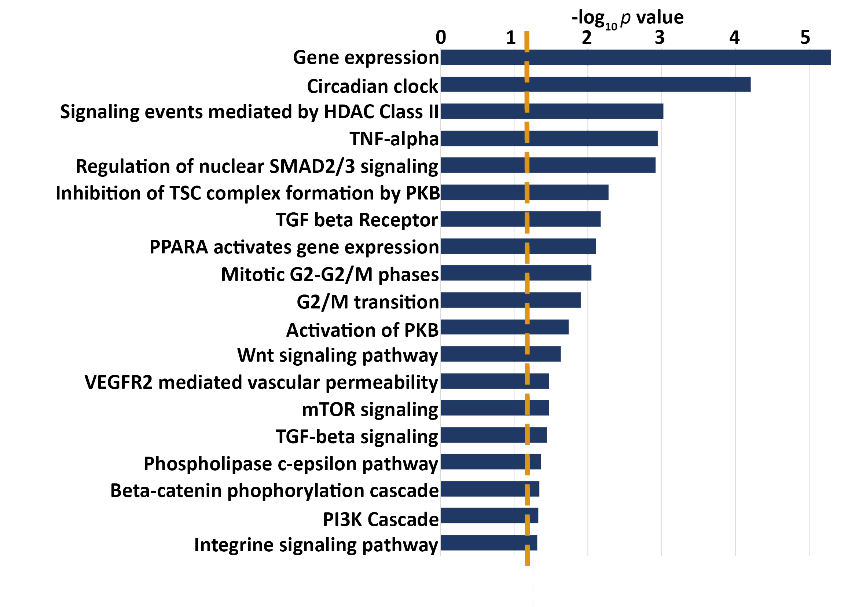
**Figure S10.** Pathway analysis of putative piRNAs mRNA targets. Pathway analysis performed for 3288 predicted targets of the PIWIL1/piRNA complex showed a significative enrichment (-log10 p value > 1.3) in cellular processes connected with gene expression and cell cycle regulation, and for signaling pathways strongly related to the CRC development and progression.

1. Supplementary Methods

*1.1 Preprocessing, alignment and annotation to small RNAs*

To identify small RNA sequences, SPORTS1.0 [[1](#_ENREF_1)] workflow was exploited; raw sequence files (.fastq files) were subjected to adapter trimming, quality control analysis and length filtering; then, clean reads were aligned to the human genome (assembly hg 38) with bowtie aligner [[2](#_ENREF_2)] (v. 1.2.2 - 12/11/2017). Default parameters were used for minimum (15 nt), maximum (45 nt) length and total number of mismatches (0mm).

Afterwards, clean reads aligned to reference were annotated to different small RNA databases. Furthermore, resulted files were processed using in-house scripts performed with R version 3.5.3 (2019-03-11) and packages from Bioconductor repository [[3](#_ENREF_3),[4](#_ENREF_4)] version 3.8. Reads aligned to reference genome and annotated to small RNA database, were integrated (“output_*_match_genome”) in one file for downstream analysis. Since reads could multi-map to different small RNAs (features) for each database in the annotation step, we considered calculating counts for small RNAs as a fractional value. Counts of small RNAs were inferred following the multi-overlap read method for features in which if a read has been annotated to more than one features, then a fractional count was reported in each overlapping feature and the sum of fractional counts assigned as the final value for each annotated feature.

For the identification of small RNAs, the following databases that have been used:

1. miRNA: miRbase [[5](#_ENREF_5)], release 22.1
2. rRNA: Sequences from the database included in SPORTS1.0 (Original source: https://www.ncbi.nlm.nih.gov/nuccore)
3. tRNA: GtRNAdb [[6](#_ENREF_6)] (High Confidence Set)
4. ncRNA: Ensembl [[7](#_ENREF_7)], release 89
5. ncRNA: Rfam [[8](#_ENREF_8),[9](#_ENREF_9)], release 12.3
6. piRNA: piRBase [[10](#_ENREF_10)], release 2.0

The databases that have been utilized are the proposed from the SPORTS1.0 tool with the only difference that we updated the version of the miRBase to 22.1 release instead of the 21 that the tool was providing and for piRNAs we used the latest release of piRBase in which it was greatly improved with new piRNA sequences.

In the page of the tool databases are provided for download. New releases from miRBase and piRBase have been downloaded from <http://www.mirbase.org/ftp.shtml> and <http://www.regulatoryrna.org/database/piRNA/download.html> respectively in fasta format in order to create the bowtie2 indexes. SPORTS1.0 performs sequential mapping against the reference genome, miRBase, rRNA, GtRNAdb, Ensembl, Rfam and finally piRBase. Not-annotated sequences which matched to reference genome where extracted from the final resulted file that includes all reads.

SPORTS1.0 parameters used are listed below:

sports.pl -i '/foo/bar/samples_directory'

-p 6

-a

-k

-x GTTCAGAGTTCTACAGTCCGACGATC

-y TGGAATTCTCGGGTGCCAAGG

-g '/UCSC/hg38/Sequence/BowtieIndex/genome'

-m '/miRBase_22.1/miRBase_22-hsa'

-w '/piRBase/piR_human'

-t '/GtRNAdb/hg38-tRNAs'

-r '/rRNA_db/human_rRNA'

-e '/Ensembl/Homo_sapiens.GRCh38.ncrna'

-f '/Rfam_12.3/Rfam-12.3-human'

-o '/foo/bar/results_dir'

*1.2 Identification of methylated piRNAs*

For the analysis of small RNAs identified after sodium periodate/β-elimination treatment, we calculated mean expression values between three replicates of treated (T) and non-treated (NT) samples; then, we estimated the log_2_ ratio between mean expression of piRNAs in treated vs non-treated samples (T/NT).

piRNAs with (log_2_(T/NT) > 2), were considered methylated; piRNAs with (-2 < log_2_(T/NT) < 2) were considered partially-methylated; piRNAs with (log_2_(T/NT) < -2) were considered non-methylated.

*1.3 piRNAs identification in the RIP-Seq experiment*

For the analysis of RIP-Seq experiment, in order to identify the enriched small-RNAs immunoprecipitated with PIWIL1 antibody, we followed the enrichment analysis strategy utilized in Tarallo et al. [[11](#_ENREF_11)] except that instead of DEseq2 [[12](#_ENREF_12)] Bioconductor package, edgeR [[13](#_ENREF_13),[14](#_ENREF_14)] and limma [[15](#_ENREF_15)] were utilized. Specifically, we considered enriched all small RNAs that presented log_2_ enrichment factor (log_2_EF) > 2 respect to input samples with false discovery rate (FDR) < 0.01. Furthermore, we excluded all enriched small RNAs that displayed enrichment in No antibody control samples respect to the input with the cutoffs mentioned above (log_2_EF, FDR). Therefore, three sets of enriched small RNAs were considered as “immunoprecipitated”: the first set included small RNAs enriched with antibody 1, the second set small RNAs purified with antibody 2 and a third set which is the intersection of the two antibodies.

*1.4 RNA-Seq analysis*

The raw .fastq file was pre-processed with fastp [[16](#_ENREF_16)] for quality control adapter trimming and filtering of low quality score bases. Next, alignment to human genome (assembly hg38) was performed using Rsubread [[17](#_ENREF_17)] package and featurecounts [[18](#_ENREF_18)] were utilized for the computation of gene read counts. Alignment was performed without allowing multi mapping and with a total number of 3 mismatches (3 mm). For the computation of gene counts, “countMultiMappingReads” was allowed but with “fraction” parameter set to “TRUE” for the fractional calculation of multi-overlapped reads. Differential expression analysis was performed with DESeq2 package.

*1.5 Pathway analyses and functional enrichment*

Pathway analysis was performed with Innate DB [[19](#_ENREF_19)], using as input the list of predicted target genes of 317 piRNAs co-purified with PIWIL1. Gene network was created using GeneMANIA [[20](#_ENREF_20)] web platform using Gene Ontology as main library.

1. Shi, J.; Ko, E.A.; Sanders, K.M.; Chen, Q.; Zhou, T. SPORTS1.0: A Tool for Annotating and Profiling Non-coding RNAs Optimized for rRNA- and tRNA-derived Small RNAs. *Genomics Proteomics Bioinformatics* **2018**, *16*, 144-151.

2. Langmead, B.; Trapnell, C.; Pop, M.; Salzberg, S.L. Ultrafast and memory-efficient alignment of short DNA sequences to the human genome. *Genome Biol* **2009**, *10*, R25.

3. Gentleman, R.C.; Carey, V.J.; Bates, D.M.; Bolstad, B.; Dettling, M.; Dudoit, S.; Ellis, B.; Gautier, L.; Ge, Y.; Gentry, J., et al. Bioconductor: open software development for computational biology and bioinformatics. *Genome Biol* **2004**, *5*, R80.

4. Huber, W.; Carey, V.J.; Gentleman, R.; Anders, S.; Carlson, M.; Carvalho, B.S.; Bravo, H.C.; Davis, S.; Gatto, L.; Girke, T., et al. Orchestrating high-throughput genomic analysis with Bioconductor. *Nat Methods* **2015**, *12*, 115-121.

5. Kozomara, A.; Birgaoanu, M.; Griffiths-Jones, S. miRBase: from microRNA sequences to function. *Nucleic Acids Res* **2019**, *47*, D155-D162.

6. Chan, P.P.; Lowe, T.M. GtRNAdb 2.0: an expanded database of transfer RNA genes identified in complete and draft genomes. *Nucleic Acids Res* **2016**, *44*, D184-189.

7. Zerbino, D.R.; Achuthan, P.; Akanni, W.; Amode, M.R.; Barrell, D.; Bhai, J.; Billis, K.; Cummins, C.; Gall, A.; Giron, C.G., et al. Ensembl 2018. *Nucleic Acids Res* **2018**, *46*, D754-D761.

8. Kalvari, I.; Argasinska, J.; Quinones-Olvera, N.; Nawrocki, E.P.; Rivas, E.; Eddy, S.R.; Bateman, A.; Finn, R.D.; Petrov, A.I. Rfam 13.0: shifting to a genome-centric resource for non-coding RNA families. *Nucleic Acids Res* **2018**, *46*, D335-D342.

9. Kalvari, I.; Nawrocki, E.P.; Argasinska, J.; Quinones-Olvera, N.; Finn, R.D.; Bateman, A.; Petrov, A.I. Non-Coding RNA Analysis Using the Rfam Database. *Curr Protoc Bioinformatics* **2018**, *62*, e51.

10. Wang, J.; Zhang, P.; Lu, Y.; Li, Y.; Zheng, Y.; Kan, Y.; Chen, R.; He, S. piRBase: a comprehensive database of piRNA sequences. *Nucleic Acids Res* **2019**, *47*, D175-D180.

11. Tarallo, R.; Giurato, G.; Bruno, G.; Ravo, M.; Rizzo, F.; Salvati, A.; Ricciardi, L.; Marchese, G.; Cordella, A.; Rocco, T., et al. The nuclear receptor ERbeta engages AGO2 in regulation of gene transcription, RNA splicing and RISC loading. *Genome Biol* **2017**, *18*, 189.

12. Love, M.I.; Huber, W.; Anders, S. Moderated estimation of fold change and dispersion for RNA-seq data with DESeq2. *Genome Biol* **2014**, *15*, 550.

13. Robinson, M.D.; McCarthy, D.J.; Smyth, G.K. edgeR: a Bioconductor package for differential expression analysis of digital gene expression data. *Bioinformatics* **2010**, *26*, 139-140.

14. McCarthy, D.J.; Chen, Y.; Smyth, G.K. Differential expression analysis of multifactor RNA-Seq experiments with respect to biological variation. *Nucleic Acids Res* **2012**, *40*, 4288-4297.

15. Ritchie, M.E.; Phipson, B.; Wu, D.; Hu, Y.; Law, C.W.; Shi, W.; Smyth, G.K. limma powers differential expression analyses for RNA-sequencing and microarray studies. *Nucleic Acids Res* **2015**, *43*, e47.

16. Chen, S.; Zhou, Y.; Chen, Y.; Gu, J. fastp: an ultra-fast all-in-one FASTQ preprocessor. *Bioinformatics* **2018**, *34*, i884-i890.

17. Liao, Y.; Smyth, G.K.; Shi, W. The R package Rsubread is easier, faster, cheaper and better for alignment and quantification of RNA sequencing reads. *Nucleic Acids Res* **2019**, *47*, e47.

18. Liao, Y.; Smyth, G.K.; Shi, W. featureCounts: an efficient general purpose program for assigning sequence reads to genomic features. *Bioinformatics* **2014**, *30*, 923-930.

19. Breuer, K.; Foroushani, A.K.; Laird, M.R.; Chen, C.; Sribnaia, A.; Lo, R.; Winsor, G.L.; Hancock, R.E.; Brinkman, F.S.; Lynn, D.J. InnateDB: systems biology of innate immunity and beyond--recent updates and continuing curation. *Nucleic Acids Res* **2013**, *41*, D1228-1233.

20. Franz, M.; Rodriguez, H.; Lopes, C.; Zuberi, K.; Montojo, J.; Bader, G.D.; Morris, Q. GeneMANIA update 2018. *Nucleic Acids Res* **2018**, *46*, W60-W64.
